# Fire Usage and Ancient Hominin Detoxification Genes: Protective Ancestral Variants Dominate While Additional Derived Risk Variants Appear in Modern Humans

**DOI:** 10.1371/journal.pone.0161102

**Published:** 2016-09-21

**Authors:** Jac M. M. J. G. Aarts, Gerrit M. Alink, Fulco Scherjon, Katharine MacDonald, Alison C. Smith, Harm Nijveen, Wil Roebroeks

**Affiliations:** 1 Faculty of Archaeology, Leiden University, Leiden, The Netherlands; 2 Laboratory of Molecular Biology, Department of Plant Sciences, Wageningen University, Wageningen, The Netherlands; 3 Division of Toxicology, Department of Agrotechnology and Food Sciences, Wageningen University, Wageningen, The Netherlands; 4 Bioinformatics Group, Department of Plant Sciences, Wageningen University, Wageningen, The Netherlands; Max Planck Institute for the Science of Human History, GERMANY

## Abstract

Studies of the defence capacity of ancient hominins against toxic substances may contribute importantly to the reconstruction of their niche, including their diets and use of fire. Fire usage implies frequent exposure to hazardous compounds from smoke and heated food, known to affect general health and fertility, probably resulting in genetic selection for improved detoxification. To investigate whether such genetic selection occurred, we investigated the alleles in Neanderthals, Denisovans and modern humans at gene polymorphisms well-known to be relevant from modern human epidemiological studies of habitual tobacco smoke exposure and mechanistic evidence. We compared these with the alleles in chimpanzees and gorillas. Neanderthal and Denisovan hominins predominantly possess gene variants conferring increased resistance to these toxic compounds. Surprisingly, we observed the same in chimpanzees and gorillas, implying that less efficient variants are derived and mainly evolved in modern humans. Less efficient variants are observable from the first early Upper Palaeolithic hunter-gatherers onwards. While not clarifying the deep history of fire use, our results highlight the long-term stability of the genes under consideration despite major changes in the hominin dietary niche. Specifically for detoxification gene variants characterised as deleterious by epidemiological studies, our results confirm the predominantly recent appearance reported for deleterious human gene variants, suggesting substantial impact of recent human population history, including pre-Holocene expansions.

## Introduction

The capacity to neutralise the adverse health effects of toxic substances is an important asset which increases “Darwinian” fitness, especially through dietary flexibility, but also by improved resistance to environmental poisons. The latter has been particularly important for the human lineage during the tens, or perhaps hundreds of thousands of years in which it enjoyed the benefits of fire use. Cooking made it possible to utilize a wider range of food resources more effectively by improving digestibility and detoxification, and fire usage enabled survival in colder regions [1–4]. While its broad range of benefits is widely recognized in palaeoanthropology, it is rarely acknowledged that fire can also provoke negative health effects including cancers and reduced reproductive success [5, 6]. This suggests that fire use might have resulted in genetic selection of new, derived genotypes conferring increased resistance to toxic fire-related compounds.

Use of biomass-fuelled fires leads to exposure to smoke toxicants, such as polycyclic aromatic hydrocarbons [7], significantly affecting human health. This is in particular evident from the vast amount of epidemiological [8–11] and biochemical [12, 13] research demonstrating the carcinogenic and adverse reproduction effects of tobacco smoke, which contains the same major toxicants as any other biomass-fuelled fire [6, 14]. Because of the extensive knowledge of the biochemical mechanisms causing the toxic effects of habitual (tobacco) smoke exposure and the role of protective variants of genes in defence against its adverse effects [6, 15, 16], we focus this study on the evolution of those genes since the divergence between the chimpanzee (unexposed reference species) and the human lineage. Genetic adaptations concerning the efficiency of dealing with poison exposure provide valuable information about lifestyle and habitat, and study of the evolution of these genes may also contribute to assessing more accurately at what point(s) regular use of fire emerged following this divergence. Current estimates of the chronology of habitual fire usage range from very early first use by *Homo erectus* in Africa at 1.8 million years ago (mya) to significantly later introduction by *H*. *sapiens* at the end of the Pleistocene [2, 17, 18]. Neanderthals and the newly discovered Denisovans, ancient hominins living in Europe and Asia between about 400 and 40 thousand years ago (kya) [19–24], were probably habitually using fire from 300 thousand years onward [17], as were their Levantine contemporaries [25], but whether they were actually able to consistently produce it, rather than using natural fire sources, is contested by some [18].

The most important toxic compounds produced during combustion of biomass [26], and through heating of food [27] are polycyclic aromatic hydrocarbons (PAHs) and heterocyclic amines (HCAs), occurring in concert when cooking on open biomass-fuelled fires. Smoke inhalation or ingestion of smoke-contaminated heated food triggers biotransformation mechanisms in the human body [28, 29]. During biotransformation of PAHs and HCAs reactive metabolites are produced, leading to the formation of bulky, covalently bound DNA adducts [30, 31], which may result in mutation upon DNA replication [32]. Moreover, exposure to PAHs induces oxidative stress [13, 29] which also has the potential to cause DNA mutation [33].

Consequently, exposure to smoke toxicants increases the risk of developing various types of cancer in humans [16] and laboratory animals [34]. More important in terms of evolution, it may also negatively affect fertility both in women [13, 35] and in men [8, 12, 36], and lead to an increase in pregnancy complications [5, 6, 37]. For genotoxic food heating products similar effects are to be expected and, consistent with this expectation, placental transfer of heterocyclic amines to the foetus [38], reduced offspring and severe teratogenic effects have been reported [39]. Significant longevity, and thus diseases of aging such as cancer, may have come late in human evolution, probably only with modern humans [40]. Therefore, in the earlier populations discussed here, the toxic effects on reproduction may have played the main role in genetic selection pressure.

Numerous epidemiological and biochemical studies of toxic compounds from tobacco smoke or heated food items are available [15, 41–45], making it possible to identify the genes involved in their detoxification and to categorize the allelic variants observed into low- and high-risk variants, the former conferring increased protection from adverse reproduction effects as compared with the latter. The low-risk variants act either by enhancing the detoxification of these compounds [15, 46], by decreasing their bioactivation (enzymatic conversion of the parent compound into a (more) toxic metabolite) [46], or by influencing the repair of damaged DNA [8, 47]. These studies document the correlation between exposure levels, DNA adduct formation, toxicity and the ensuing health risk, and modulation of that risk by genotype. A well-known example is the increased cancer risk in smokers carrying a deletion of the entire *GSTM1* gene [15], but adverse reproduction effects have been frequently reported as well [8–11, 48, 49]. These epidemiological data demonstrate that reproductive health risks were likely associated with biomass-fuelled open fires in prehistoric times, which is further substantiated by contemporary studies on the effects of smoke exposure [5, 6, 37]. Focusing on gene polymorphisms with an established effect on susceptibility to smoke and food heating toxicants may therefore yield important information on the evolutionary history of human detoxification capacity.

The genes involved in neutralising toxic smoke components and food heating products mostly display a wide range of target compounds and are representative for the major detoxification capacities [50, 51] and their evolution. They act in concert within well-characterised detoxification pathways [11]. Therefore, these genes are expected to evolve consistently into the direction of increased detoxification efficiency if under selection pressure from a major toxic challenge such as smoke exposure. The availability of a number of ancient hominin and other primate genomes, including a high-coverage genome of a Neanderthal and a Denisovan hominin [22–24, 52], multiple human genomes from the early Upper Palaeolithic (45 kya) to the present day [53–58], and the genome-wide variation among 2504 present-day humans [59], makes analysis of the evolution of detoxification capacity in the human lineage now possible, even though only a few ancient genomes are presently known.

We postulate that, during human evolution, selection pressure towards increased resistance against fire-related toxicants was generated once humans started to use fire on a routine basis. This adaptive evolutionary model also implies that these protective variants are expected to be new beneficial mutants that will be derived alleles as compared to non-exposed great apes carrying the less efficient ancestral variant. Once present, these new beneficial variants will be under positive selection resulting in adaptation towards improved defence. In this study we explore the available genomic information for evidence of such genetic adaptations and discuss the implications of the results for the evolutionary history of human detoxification capacity. Although our results turned out to be non-conclusive regarding the time depth of fire usage, they opened a new, surprising perspective on the evolution of the involved detoxification capacities, the implications of which are discussed.

## Methods

### Determination of genetic variants in ancient hominins and Pleistocene and Holocene humans

The genetic variants occurring in Neanderthal and Denisovan genomes were derived from two high-coverage genomes, one from a Denisovan [22, 23] and one from a Neanderthal individual [24], both found at Denisova cave in Siberia, and estimated to be at least 50 ky old [60], and hence predating the earliest known Upper Palaeolithic human fossils in Eurasia. In addition, six low-coverage Neanderthal genomes derived from fossil bones found at various European sites [52] and dated between 38 and 70 ky old were analysed. The genetic variants occurring in Upper Palaeolithic hunter-gatherers were derived from genomes from two individuals, found at Ust’-Ishim and Mal’ta (MA-1) in Siberia, and respectively dating to around 45 and 24 kya [53, 55], and one from North America (Anzick-1), dating to around 12.6 kya [56]. The Holocene examples include a Neolithic (NE1; 7.2 kya) and a Bronze Age (BR2; 3.2 kya) individual from Hungary [54], an approximately 4,000 year old Palaeo-eskimo (Saqqaq) [58], and a 100-year old Aboriginal Australian [57].

### Comparison with modern human and great ape genetic variants

Variant comparisons were performed using the UCSC Genome Browser, or variants were retrieved from the NCBI Sequence Read Archive. The chimpanzee variants were taken from the chimpanzee reference genome [61], and from the PanMap project reporting the genomic sequences of ten chimpanzee individuals [62]. Gorilla variants were derived from the gorilla reference genome [63]. Allele frequencies of polymorphic sites among various modern human ethnic groups were retrieved from the 1000 Genomes Project data [64], complemented with data from the HapMap Project [65]. The variant data of the ethnic individuals were derived from Meyer *et al*. (2012) [23], except for the Yoruba trio, which were retrieved from the March 2010 release of the 1000 Genomes Project [66].

### Analysis of complete protein-coding regions

To analyse the complete protein-coding sequence of the *CYP1A1* and *CYP1B1* genes in ancient hominins, their genomic sequence was extracted from the corresponding Variant Call Format (VCF) file and all positions in translated sequences that were different from the human reference genome were filtered out using Excel.

### Statistical aspects

The distribution of the number of low-/high-risk alleles within a global population of 2504 present-day individuals of the 1000 Genome Project [59] was determined to test the significance of the different predominance of the low-risk detoxification gene variants observed in ancient hominins and anatomically modern humans. Details in Section B of S1 Text.

See S1 Text (Supporting Information) for detailed procedure information.

## Results

### Gene polymorphisms affecting reproduction in modern humans in interaction with smoking behavior

Nineteen genes relevant for detoxification of toxic smoke components and food heating products were identified on the basis of biochemical knowledge of their detoxification mechanism (Tables 1–3), and by screening the epidemiological literature for studies reporting an interactive effect of exposure to these poison categories and polymorphisms in a relevant gene, acting on health parameters affecting reproductive success (details in S1 Table).

**Table 1:** Polymorphisms for which the low-risk gene variant observed in Neanderthal and/or Denisovan is the ancestral allele. Column headers: Nea = Neanderthal; Den = Denisovan; Chimp = Chimpanzee; Gor = Gorilla; Ust’-Ishim/Mal’ta (MA-1)/Anzick-1 = Siberian/Siberian/North-American pre-Holocene hunter-gatherer; NE1/BR2 = Neolithic/Bronze Age Hungarian individual; Saqqaq = Palaeo-eskimo; Aus = Aboriginal Australian. Cell shading: light grey = low-risk ancestral variant; dark-grey = high-risk derived variant.

Gene	Function^1^^)^	Extant Humans				AncientHominins		Great Apes			Modern Humans													
		Polymorphism		Low^3^^)^	High^3^^)^	Nea	Den	Chimp		Gor	Ust'-Ishim		MA-1		Anzick-1		NE1		Saqqaq		BR2		Aus	
		RefSNP Number	HGVS Name^2^^)^								(45 kya)		(24 kya)		(12 kya)		(7.2 kya)		(3.9 kya)		(3.2 kya)		(0.1 kya)	
*AHR1*	Regulation	rs2066853	p.Arg554Lys	A	G	A	A	A		A	A	G	A		G		G		G		G		─	
* *		rs2282885	c.66-3946A>G	A	G	A	A	A		A	A		A		A		A	G	A	G	A	G	A	
*AHRR1*	Regulation	rs2292596	p.Pro189Ala	C	G	C	C	C		C	C	G	C	G	C		G		C	G	C	G	C	G
*CYP1A1*	Detox. 1	rs4646903	c.*1189T>C	T	C	T	T	T		T	T		─		T	C	T		T		T	C	C	
* *		rs1048943	p.Ile462Val	A	G	A	A	A		A	A		A		A	G	A		A		A	G	A	
*CYP1B1*	Detox. 1	rs1056836	p.Leu432Val	G	C	G	G	G		G	G		C		C		C		G	C	C		C	
*EPHX1*	Detox. 1	rs1051740	p.Tyr113His	T	C	T	T	T		T	T		C		T		T	C	T	C	T		T	
*EPHX2*	Detox. 1	rs1042064	c.*93T>C	C	T	C	C	C		C	C	T	C		T		T		C		T		T	
*GSTA4*	Detox. 2	rs316133	c.415-48C>G	G	C	C	G	G		G	G		─		G		G		G		G		─	
* *		rs3756980	c.139+176T>C	T	C	C	T	T		T	T		T		T		T		T		T		T	
*GSTM1*	Detox. 2	-	Null^4^^)^	WT	Null	WT	WT	WT		WT	WT		WT		Null		WT		Null		WT		Null	
*GSTP1*		rs1138272	p.Ala114Val	C	T	C	C	C		C	C		C		C		C		C		C		C	
* *		rs762803	c.232+13C>A	C	A	C	C	C		C	C	A	C		C	A	A		C		C		C	
*GSTT1*	Detox. 2	-	Null^4^^)^	WT	Null	WT	WT	WT		WT	WT		WT		WT		Null?^5^^)^		Null		WT		Null	
*NAT1*	Detox. 2		c.*215A>T	T	A	T	A	T	Diff.^6^^)^	Del.^6^^)^	T		T		A		T	A	T		T		─	
		rs4986782	p.Arg187Gln	G	A	G	G	G		G	G		G		G		G		G		G		─	
* *		rs5030839	p.Arg187Ter	C	T	C	C	C		C	C		C		C		C		C	T	C		─	
* *		rs56379106	p.Arg64Trp	C	T	C	C	C		C	C		C		C		C		C		C		C	
* *		rs56318881	p.Arg33Ter	C	T	C	C	C		C	C		C		C		C		C		C		C	
* *		rs56172717	p.Asp251Val	A	T	A	A	A		A	A		A		A		A		A		A		─	
*NAT2*	Detox. 2	rs1801280	p.Ile114Thr	T	C	T	T	T		T	T		T		T		T	C	T		T		T	
* *		rs1799930	p.Arg197Gln	G	A	G	G	G		G	G		G		G	A	G		G		A		─	
*CAT1*	Anti-ox.	rs1001179	c.-330C>T	C	T	C	C	C		C	C	T	T		C		C		C		C		C	
*SOD2*	Anti-ox.	rs4880	p.Ala16Val	C	T	C	C	C		C	C	T	T		C	T	C		C		T		C	T
* *		rs5746136	c.*441G>A	G	A	G	G	G		G	G	A	A		G		G		G		G		G	
*XPA1*	Repair	rs1800975	c.-4A>G	G	A	G	G	G		G	G	A	G		G	A	G	A	G		G	A	G	A

1 Detox 1 = Detoxification phase 1; Detox 2 = Detoxification phase 2; Anti-ox = Oxidative stress management; Regulation = Regulation of detoxification gene expression; Repair = Repair of DNA damage.

2 SNP nomenclature as recommended by the Human Genome Variation Society (HGVS) (http://www.hgvs.org/mutnomen/recs.html).

3 Allele associated with a relatively low-risk, respectively, high-risk of adverse reproduction effects based on epidemiological or biochemical studies (details in S1 Text of the Supporting Information).

4 Null variant has a deletion of the entire gene sequence; WT = wild-type allele.

5 Most likely Null for GSTT1, since only reads observed in intron regions, not in exon regions.

6 This A/T polymorphism is the 3'-terminal base of a tandem repeat of four AAT elements that is found full length in modern humans, Neanderthals, and the Denisovan, but is variable in length in chimpanzees (0–4 AAT elements, following position 14,328,777 in assembly CSAC 2.1.4/panTro4). Only the full length 4 x AAT variant encompasses the position corresponding to human polymorphism rs1057126. Diff. indicates that some of the PanMap chimpanzees are clearly or possibly (indicated by? in S2 Table) heterozygous for variants encompassing 0–4 AAT elements. Del. indicates that gorilla misses all 4 AAT elements (= 0 x AAT).

**Table 2:** Polymorphisms for which the low-risk gene variant observed in Neanderthal and/or Denisovan is a derived allele. Column headers and cell shading as for Table 1.

Gene	Function^1^^)^	Extant Humans				AncientHominins			Great Apes		Modern Humans											
		Polymorphism^2^^)^		Low^3^^)^	High^3^^)^	Nea		Den	Chimp	Gor	Ust'-Ishim		MA-1	Anzick-1		NE1		Saqqaq		BR2	Aus	
		RefSNP Number	HGVS Name^2^^)^								(45 kya)		(24 kya)	(12 kya)		(7.2 kya)		(3.9 kya)		(3.2 kya)	(0.1 kya)	
*EPHX1*	Detox 1	rs2234922	p.His139Arg	A	G	A		A	G	G	A		─	A		A		A		G	A	G
*GSTP1*	Detox 2	rs1695	p.Ile105Val	A	G	A^4^^)^	G	A	G	G	A	G	A	A	G	G		A		A	A	
*ERCC1*	Repair	rs3212986	c.*197G>T	G	T	G		G	T	T	G		G	G		G	T	G	T	G	G	

1. Detox 1 = Detoxification phase 1; Detox 2 = Detoxification phase 2; Repair = Repair of DNA damage.

2. SNP nomenclature as recommended by the Human Genome Variation Society (HGVS) (http://www.hgvs.org/mutnomen/recs.html).

3. Allele associated with a relatively low-risk, respectively, high-risk of adverse reproduction effects based on epidemiological or biochemical studies (details in S1 Text of the Supporting Information).

4) Neanderthal individual Vi33.26 is possibly heterozygous low-/high-risk (A/G) based on a single, high-quality read of each type; the high-coverage Altai Neanderthal and possibly Vi33.25 (one high-quality G read) are homozygous for the high-risk G allele.

**Table 3:** Polymorphisms for which the high-risk gene variant was observed in both Neanderthal and Denisovan. Column headers and cell shading as for Table 1. Ancestral variants in the ancient hominin and human lineages are indicated by a thick-lined black box.

Gene	Function^1^^)^	Extant Humans				AncientHominins		Great Apes		Modern Humans											
		Polymorphism^2^^)^		Low^3^^)^	High^3^^)^	Nea	Den	Chimp	Gor	Ust'-Ishim		MA-1	Anzick-1		NE1		Saqqaq		BR2		Aus
		RefSNP Number	HGVS Name^2^^)^							(45 kya)		(24 kya)	(12 kya)		(7.2 kya)		(3.9 kya)		(3.2 kya)		(0.1 kya)
*AHR1*	Regulation	-^4^^)^	p.Val381Ala^4^^)^	T	C	C	C	C	C	T		─	T		T		T	A^5^^)^	T		─
*CYP1A1*	Detox 1	rs4646421	c.-26-728C>T	T	C	C	C	C	C	C		T	T	C	C		C		T	C	T
*NAT1*	Detox 2	rs15561	c.*222A>C	C	A	A	A	A	A	C	A	C	A		C	A	C		C		─
*SULT1A1*	Detox 2	rs9282861	p.Arg213His	A	G	G	G	G	G	G		─	─		G		─		G		G
*UGT1A7*	Detox 2	rs17868323/rs17863778/rs17868324	T387G/C391A/G392A^6^^)^	T/C/G	G/A/A	G/A/A	G/A/A	G/A/A	G/A/A	G/A/A		G/A/A	T/C/G		G/A/A		─		G/A/A		─
* *		rs11692021	p.Trp208Arg	T	C	C	C	T	T	T	C	C	T		C		T		T	C	T
*HIF1A1*	Anti-ox	rs2301113	c.1609-675C>A	A	C	C	C	C	T	C		A	A	C	A		A		A	C	A

1. Detox 1 = Detoxification phase 1; Detox 2 = Detoxification phase 2; Anti-ox = Oxidative stress management; Regulation = Regulation of detoxification gene expression.

2. SNP nomenclature as recommended by the Human Genome Variation Society (HGVS) (http://www.hgvs.org/mutnomen/recs.html).

3. Allele associated with a relatively low-risk, respectively, high-risk of adverse reproduction effects based on epidemiological or biochemical studies (details in S1 Text of the Supporting Information).

4) Up to now only the AHR protein variant with Val at position381 has been observed in present-day humans and its risk-evaluation is based on laboratory animal data. No RefSNP code available.

5) On this position of the Saqqaq genome one A read was observed, which was however of comparable quality as the two T reads on this position.

6) HGVS nomenclature: p.Asn129Lys / p.Arg131 = / p.Arg131Gln.

### The majority of detoxification-related genes in ancient hominins are in the ancestral state, predominantly being the protective variant

For the majority of gene polymorphisms studied (29 out of 36) we found that both the Altai Neanderthal and Denisovan genomes were still homozygous for the ancestral variant observed in chimpanzees and gorilla (Tables 1–3; and S2 Table). In 23 out of these 29 cases this ancestral allele turned out to be the protective variant conferring a decreased reproductive health risk from exposure to genotoxic combustion and food heating products (Table 1). In addition, there are two cases in which only the Denisovan ancient hominin species was carrying the ancestral (protective) allele (Table 1), and also two cases where only the Neanderthals display the ancestral state (Table 1: c.*215A>T in *NAT1* homozygous ancestral low-risk; Table 2: p.Ile105Val in *GSTP1* heterozygous ancestral high-risk/derived low risk in the Vi33.26 Neanderthal). Together, in 33 out of 36 cases at least one of the ancient hominin species studied, Neanderthal or Denisovan, displays the ancestral allele (complete overview in S2 Table). The amount of sequence information obtained from the low-coverage genomes was too low to allow unequivocal determination of their zygosity, but we found only one instance (p.Ile105Val in *GSTP1*) where one of the low-coverage genomes clearly displayed another allele than the Altai Neanderthal (in the heterozygous state) (see S2 Table). Therefore the low-coverage genome data in general corroborated the representativeness of the Altai Neanderthal genome. That the ancient hominins predominantly carried the ancestral alleles suggests that there was no need to adapt their detoxification capacity, which might still be in agreement with our working hypothesis, if regular use of fire did not start in the ancestral line of the ancient hominins studied here. However, it is unexpected and contradictory to our working hypothesis, that in the majority of cases (at 26 out of 33 loci being ancestral in any of both ancient hominin species), the ancestral allele turns out to be the allele that is more protective against (tobacco) smoke- and food heating-related toxins in modern humans (Tables 1 and 3). For *CYP1B1* this tendency was also confirmed on the haplotype level, as all informative Neanderthals, chimpanzees, and the Denisovan were found to carry the ancestral haplotype (Gly48, Ser119, Val432, Asn453), the haplotype with the highest catalytic efficiency observed in modern humans and a 3.4 times higher catalytic efficiency than the modern human wild-type [67]. Altogether, our observations imply that for the majority of “smoke-protective” gene variants it is not possible to demonstrate genetic adaptation, as they appear to be the ancestral alleles.

### The emergence of derived variants in ancient hominins

For 7 out of 36 loci we observed a derived allele in Neanderthal and/or Denisovan. (Tables 1–3, S2 Table). In 3 cases this derived variant is the one associated with relatively low-risk for adverse effects of tobacco smoking in modern humans (Table 2). In 4 cases the derived allele emerging is the high-risk variant (Tables 1 and 3). Since these data did not show a clear bias for the appearance of either new low-risk or high-risk alleles (3 against 4) in the Neanderthal and Denisovan lineages, they do not present a clear signature of positive selection that could be driven by smoke exposure, consistent with the conclusion that no major adaptation in this direction occurred in this group of genes as compared to great apes.

### Low-risk versus high-risk alleles in ancient hominins

**Fig 1:**
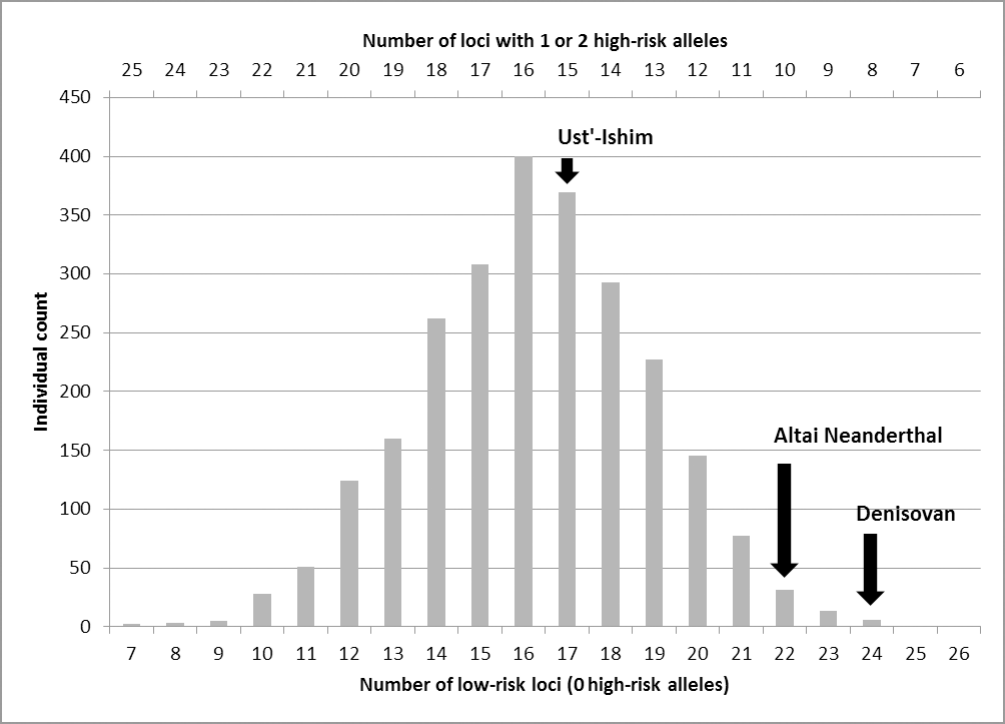
Distribution of the number of low-risk loci (0 high-risk alleles) and loci with 1 or 2 high-risk alleles within the global population of the 1000 Genome Project [59]. The average number of loci carrying 0/(1 or 2) high-risk alleles was 16/16 and coincided with the median of this distribution. The relative position of the Altai Neanderthal and the Denisovan hominin high-coverage genomes are indicated, as well as the genome of the oldest available anatomically modern human, that of a 45,000 year old individual from Ust’-Ishim. Three SNPs (rs2292596, rs56318881, and rs9282861) analysed in this study were not covered by the 1000 Genome Project variant data and were therefore not included in this analysis. For each of the GSTM1 and GSTT1 loci in the ancient hominin genomes, being possibly homozygous or heterozygous low-risk (S2 Table), a contribution of 1 high-risk allele was conservatively counted in. Details in Section B in S1 Text.

Out of the 35 loci studied (p.Val381Ala in *AHR1* was not included here because its risk status was not confirmed in a human background), 25 carried the protective (low-risk) allele in both Neanderthal and Denisovan hominins, whereas the high-risk variant was found consistently in both species at only 6 of these loci (Table 3) (note that all 35 loci were found homozygous; S2 Table). Furthermore, there were 4 cases where the results were contradictory in the sense that either the Denisovan or the Neanderthals had the high-risk allele, whereas the other hominin species studied showed the protective allele (Tables 1 and 2), or both the high- and low-risk allele seemed to occur (p.Ile105Val in *GSTP1* in the Neanderthals; Table 2). Per separate locus, we have only information from a very limited number of ancient hominin individuals. However, in all cases where the low-risk allele was found as the ancestral allele, it is highly unlikely that the high-risk allele would also be present in the ancient hominin population at a considerable frequency for reasons explained in detail in Section H of S1 Text. Moreover, we compared the two representatives from different ancient hominin species (Altai Neanderthal and Denisovan) to the global population of the 1000 Genomes Project (Fig 1), which showed that the probability of finding a pair of individuals with an equal or more pronounced predominance of the low-risk alleles in the present-day human population is very low (about 1.6%). These arguments corroborate that it is very unlikely that the derived high-risk alleles would have reached a substantial frequency without appearing in our ancient hominin sample. Altogether, we conclude that our data support a predominance of the low-risk over the high-risk alleles in Neanderthal and Denisovan hominins over the 35 polymorphic loci within a group of 19 detoxification genes studied here, where in the present-day human population the high-risk allele is always found next to the low-risk allele.

### Neanderthals and Denisovans carry the ancestral variant of AHR, a key regulator of detoxification genes

The *AHR1* gene encodes the aryl hydrocarbon receptor (AHR), the key regulator of *CYP1A1*, *CYP1B1*, and other important phase 1 and 2 detoxification enzyme genes. The modern human AHR has a valine residue at position 381 in the ligand-binding domain, resulting in lower binding affinity for prototype AHR agonists [68], a shifted ligand selectivity [69, 70], transcription activation agonist efficiency [71] and target gene range [72] as compared to the mouse homolog *Ahr*, which carries Ala at homologous position 375 [68]. Surprisingly, both Neanderthals (Altai and Vi33.16; S2 Table) and the Denisovan carry the high affinity *AHR1* gene with an Ala residue at position 381 (Table 3), and are thus again similar to the chimpanzee and gorilla in this respect, and different from modern humans.

### In modern humans high-risk variants are already present in early Upper Palaeolithic hunter-gatherers

At all 25 loci where exclusively the low-risk allele was observed in Neanderthals and the Denisovan, both allelic variants, the low-risk as well as the high-risk, were observed in present-day humans, suggesting an increase in number and/or frequency of the high risk alleles at these loci. Therefore, we determined the distribution of the number of loci carrying zero or at least one high-risk alleles within the global population of 2504 modern humans studied by the 1000 Genomes Project Consortium [59]. Comparing the Altai Neanderthal and the Denisovan with this distribution showed that the Altai Neanderthal with 10 loci carrying 1 or 2 high-risk alleles, is at the far end of the distribution, with only 2.0% of contemporary humans having an equal or lower number of such loci, over the 32 loci that were covered by the 1000 Genomes genetic variant data (Fig 1). The Denisovan, with 8 of such loci, even falls at the extreme end of the contemporary human distribution (0.24% has also 8, none has less).

The earliest sequenced anatomically modern human genome from an early Upper Palaeolithic hunter-gatherer from Ust’-Ishim (western Siberia), dated to approximately 45,000 years ago [53], is clearly shifted towards a higher number of loci carrying 1 or 2 high-risk alleles (Fig 1), although this individual, with 15 of such loci, is still slightly below the median of the contemporary human population (Fig 1). Indeed, Tables 1 and 2 specify, that at eight loci high-risk variants are observed in the Ust’-Ishim individual that were not observed in the studied Neanderthal, Denisovan, chimpanzee or gorilla genomes. Because all of these eight loci are heterozygous high-/low-risk loci, the increase is less pronounced at the allele level (see also Section B of S1 Text). There is a distinctive increase in the number of high-risk alleles within the Group A loci of the Ust’-Ishim genome as compared to the ancient hominin genomes (details in Section I of S1 Text). An overall increase in number over all loci investigated could not be unambiguously demonstrated, although the data suggest a small increase, most probably through the emergence of new derived variants. In view of the high global allele frequencies attained by a number of high-risk alleles in present-day humans (Tables C-E in S1 Text), an increase in the frequency of many derived high-risk alleles in Ust’-Ishim as compared to Altai Neanderthal and Denisovan becomes more likely and cannot be excluded as well. In younger genomes, some derived high-risk variants present in older genomes are absent, but also some derived low-risk variants, and there is no clear indication that the frequency of occurrence of high-risk variants gradually increases in AMHs over time (Tables 1–3; S2 Table). Altogether, these data indicate that in the evolutionary lineage leading to AMHs more high-risk alleles might have emerged than on the lineage leading to Neanderthal and Denisovan.

In present-day humans, however, the high-risk variants occur at all 35 loci investigated, attain global allele frequencies up to 81%, exceed the 5% limit to become a common variation at 29 of these 35 loci, and are the major allele at 11 loci (Tables C-E in S1 Text; S2 Table). For a group of 21 loci found ancestral and homozygous low-risk in both high-coverage ancient genomes studied (Group A) and less clearly for the remaining 14 loci, our data indicate an increase in the number of high-risk alleles in present-day humans as compared to Neanderthals and the Denisovan (details in Section I of S1 Text). In addition, the high contemporary global allele frequencies for the mainly derived high-risk alleles provide a strong indication for an overall increase in frequency in modern humans as compared to Neanderthal and Denisovan hominins (Tables C-E in S1 Text). The Ust’-Ishim data are consistent with an early start of this increase in number and frequency before 45 kya (Tables 1–3).

### Geographical distribution

Both high- and low-risk variants of the polymorphisms studied are present in many contemporary populations throughout the world (S2 Table). Overall, there are no strong differences in the number of high-risk variants observed in various populations and individuals all over the world, although for specific gene polymorphisms only one allele is observed in particular regions. However, one large-scale regional difference suggests itself: three polymorphisms can be identified (AHR1/c.66-3946A>G; CYP1A1/p.Ile462Val; GSTP1/p.Ala114Val) for which the high-risk variant is (almost) absent (range 0–2%) in Africa, while both high- and low-risk variants are observed outside Africa (range of high-risk allele frequency (3–40%). Grouping the results in terms of the function of the gene product in detoxification did not strengthen any pattern or indicate any additional pattern.

## Discussion

For the majority of gene polymorphisms (29 out of 35) affecting sensitivity to the adverse effects of genotoxic combustion and food heating products we observed that Neanderthal and/or Denisovan hominins carried the protective (low-risk) allele. Since we analysed 35 loci in 19 functionally related genes in at least 2 ancient hominin genomes (the high-coverage Denisovan and Altai Neanderthal, and up to 6 low-coverage Neanderthal genomes) coincidence because of the low number of ancient hominin genomes analysed can be excluded as the explanation of the significant genetic pattern observed. Four population-genetic principles provide support to this conclusion (details in Supporting Information, Sections H-I of S1 Text): (I) The high-risk variants were found to be derived in most cases, and derived alleles tend to occur at lower frequencies than ancestral alleles [73]. (II) Neanderthals and Denisovans have experienced population bottle-necks [74] and (III) display an unusually low genetic diversity [23] or are very much inbred [24], and (IV) are both mostly homozygous at the loci studied here (S2 Table). Each of these arguments makes it less likely that another allele would have existed at substantial frequency next to the ancestral low-risk alleles predominantly observed in the ancient hominin populations studied here. This is also corroborated by the fact that, compared with the distribution in modern humans, the ancient hominins are outliers regarding their low number of loci displaying a high-risk allele (Fig 1; see also Section B of S1 Text). As such, this pattern would be consistent with regular use of fire, since the postulated genetic selection pressure, driven by the adverse effect of fire-borne toxicants on reproductive success, would favour an increase in frequency of the low-risk genotypes. However, in 26 out of 29 cases the low-risk gene variant in ancient hominins appears to be the ancestral allele also found in chimpanzee and/or gorilla (Tables 1 and 2). This is contradictory to our hypothesis, that the low-risk alleles are expected to be newly derived alleles. The 3 cases where we did observe a new derived protective allele (Table 2), to the best of our knowledge, are not exceptional for biological reasons and are therefore outweighed by the 26 cases of ancestry. That the protective variants are ancestral means that no adaptation of these genes could be demonstrated and does not allow us to conclude to what extent genetic selection pressure plays a role in the high prevalence of low-risk alleles in ancient hominins. Hominins have undergone major changes in diet and habitat since the human and chimpanzee lineages split, minimally 7–8 million years ago [75]. The fact that, nevertheless, the majority of the gene loci studied here did not diversify by genetic drift since the gorilla and (chimpanzee–hominin) lineages diverged is consistent with the sustained functional importance of these protective gene variants, and it is possible that genetic selection plays a role in their maintenance [76, 77].

Previous studies demonstrated that the deviation of Neanderthal genomes from the modern human reference genome generally falls within the variation among modern humans [52]. Over a total of about 19,000 human genes [78] only a few hundred prominent non-synonymous substitutions were found in which the modern human variant has reached a prevalence above 90% [24]. Consistent with these observations, the Ala381 variant of *AHR1* was among the polymorphisms studied, the only variant found in ancient hominins which was not covered by modern human genetic diversity. Moreover, we confirmed that the coding region deviation in the CYP1A1 and CYP1B1 genes of ancient hominins, two key players in the detoxification of PAHs, falls within the range observed in modern humans (Section E of S1 Text; S3 and S4 Tables). These observations by us and other research groups confirmed the low sequence divergence between modern and ancient hominins and justify extrapolation of the classification of modern detoxification gene variants into high- and low-risk to the ancient hominin genomes studied here.

For all 35 risk-classifiable loci studied here, a high risk variant was observed next to the low-risk variant in modern humans, whereas the studied genomes of both ancient hominin species displayed a predominance of the low-risk alleles. In addition, the pair of high-coverage genomes studied were clear outliers when compared to the present-day human population, in particular regarding their low number of loci carrying 1 or 2 high-risk alleles (Fig 1). implying that ancient hominins very probably had a distribution shifted towards a lower number of such loci. Therefore, we argue that the presented data indicate an overall increase in high-risk allele numbers along the modern human lineage and provide strong evidence for a concomitant increase in certain allele frequencies, which is also supported by the substantial allele frequencies, up to the 80% range, often attained by the high-risk variants in contemporary humans (see Tables C-E in S1 Text), suggesting a shift of the population median towards a lower efficiency of the encoded detoxification capacities.

Since the use of fire must have become common practice at some point during the evolution of modern humans, the unexpected recent relaxation in the defence against toxic smoke components may suggest that the genetic variants studied were less important than expected. However, in view of the epidemiology of tobacco smoking a more likely explanation is that, compared with earlier hominins and great apes, the total toxic burden decreased recently, in spite of the additional challenge provided by the exposure to smoke. It is tempting to speculate that this could be due to the increased detoxification of foods by cooking, and/or the decreased exposure to UV-induced hazardous compounds (discussed below) due to cultural adaptations, such as clothing and housing. Alternatively, the increase in high-risk detoxification gene variants could be part of a larger phenomenon. One recent study found that a high proportion of single nucleotide variants are rare, and of those predicted to be deleterious 86% are estimated to have arisen during the last 10,000–5,000 years [79]. This could reflect one or more recent population expansions, particularly during the Neolithic [79]. Population expansion can cause an increase in the proportion of rare and deleterious variants, because the effect of purifying selection on less favourable variants is reduced [80]. Furthermore, fewer predicted deleterious alleles were observed in African Americans compared with European Americans, a geographical pattern that is consistent with weaker purifying selection due to the “Out of Africa” dispersal [80]. The presence of high-risk variants in the oldest modern human in our sample, (from Ust’-Ishim in Siberia), suggests that niche changes associated with the introduction of agriculture are not responsible for the increase in high-risk variants in modern humans. Further insight into this issue will come from future analyses of a larger sample of prehistoric human ancient DNA. The fact that we found three polymorphisms for which the high-risk variant is observed outside Africa, but is (almost) absent in Africa could be read as supporting the role of an ‘Out of Africa’ or subsequent dispersal. However, clear patterns do not emerge from the geographic distribution data visualized in S2 Table.

At the other end of the chronological spectrum, our study raises the question of the evolutionary origins of the low-risk variants. Answering this question will require more extensive phylogenetic comparison, which is complicated by the greater difficulty of establishing similarity in the haplotype context of the alleles. Since chimpanzees and gorillas are not exposed to smoke and cooked foods, there must be another reason for the early predominance of the low-risk alleles. One possible factor is that the diet of extant great apes contains deleterious compounds that deploy biotransformation mechanisms similar to toxic combustion products. Many plant species contain substantial levels of flavonoids [81] and other polyphenols [82], which play a role in defence against pathogens. Particularly at higher doses, these compounds may induce oxidative stress when metabolised by the animal body [83] supporting the argument that these detoxification capacities may have been required to efficiently neutralise them. A second factor may be that under the influence of UV light dioxin-like compounds are formed from the amino acid tryptophan, for example 6-formylindolo[3,2-b]carbazole (FICZ) [84, 85]. These photo-oxidation products turn out to be highly potent activators of AHR, the key mediator of dioxin-like toxicity (including PAHs) [86]. [87], and this appears to be evolutionarily conserved [88, 89]. FICZ is formed in vivo in humans in significant quantities [84, 85] and besides photo-oxidation, plant food components such as indole-3-carbinol may also give rise to similar indolocarbazole compounds in the acidic environment of the stomach [90]. Moreover, during photo-oxidation of tryptophan oxygen radicals are formed [91] leading to oxidative stress and DNA damage [92], and AHR activation by itself was observed to produce oxidative stress [93, 94]. Altogether these observations provide compelling evidence that indolocarbazoles are potentially hazardous metabolites. On the other hand, these products were found to play a role as endogenous AHR-mediated regulators of important physiological processes such as circadian rhythms and adaptation to UV exposure [95, 96]. Apparently, they need to be kept at a low, physiologically balanced level. Since their fur is relatively thin [97], this might be an important reason why chimpanzees and gorillas need an efficient CYP1A1 enzyme variant and related biotransformation capacities, as well as efficient gene variants to combat oxidative stress. Since Sandel *et al*. [97] found a significant negative correlation between body size and hair density in primates, this would suggest that the low-risk variants are adaptive in the large-bodied great apes, and plausibly for that reason be ancestral in hominins (already present in a chimpanzee-sized common ancestor). Denisovans and Neanderthals were certainly exposed to sunlight, were eating wild plant foods, while also eating more animal products [98], and may also have had to deal with additional toxicants from fire. Circumstances were probably not much different for early Upper Palaeolithic hunter-gatherers, suggesting that other explanations than environmental factors for changes in the modern human lineage have to be found.

## Conclusion

We studied the evolution of detoxification capacity by determining the genetic variants found in Neanderthal and Denisovan hominins for genes known to have an impact on the sensitivity to reproductive impairment, when exposed to genotoxic combustion and food heating products. We hypothesized that selection occurred in favour of lower risk genotypes in the human lineage after the most recent common ancestor of humans and great apes. We observed a predominance of protective alleles not only in Neanderthal and Denisovan hominins, but also in chimpanzees, showing that these protective variants are mainly ancestral. Therefore, our results turned out to be non-conclusive regarding the time depth of fire usage and our hypothesis did not find any support in our study. However, our results do show that high-risk detoxification gene alleles became more numerous in modern humans, an increase which may have already started in early Upper Palaeolithic hunter-gatherers, suggesting that it preceded the major behavioural and dietary changes of the Neolithic. This finding supports previous studies [79, 80, 99] indicating that increases in population size led to the accumulation of alleles with a negative health effect in human populations worldwide. Apparently, extant human smoke detoxification capacity is to a large degree hitchhiking on detoxification capacities developed in our primate past which had nothing to do with fire. This ancestral primate capacity is possibly directed towards the detoxification of plant polyphenols and indolocarbazole compounds originating from the diet and UV-mediated oxidation of tryptophan in the skin. Hominins must have undergone major dietary changes adapting to different habitats in the millions of years of dietary and niche change since our last common ancestor with chimpanzees. It is surprising that these changes apparently did not lead to major changes in the genes under consideration here, an inference that can be tested more robustly when more high coverage Pleistocene genomes become available. Finally, our study provides unexpected insights into the health risks facing our ancestors as well as extant humans and raises some interesting questions for future research, in particular regarding the distant origins and driving forces of the evolution of detoxification mechanisms, and the role of multiple recent population expansions.

## Supporting Information

S1 Table Genes and polymorphisms relevant for defence against toxic smoke components and food heating products analysed in this study. (DOCX)

S2 Table Genetic variants of the studied polymorphisms from modern humans that were observed in Neanderthal and Denisovan hominins, ancient anatomically modern humans, and chimpanzees and gorilla, as well as in global ethnic groups and individuals. (XLSX)

S3 Table CYP1A1 cDNA positions that are different between chimpanzee and human, and the corresponding alleles of the human, chimpanzee and gorilla reference genomes, and of the PanMap chimpanzees. (XLSX)

S4 Table CYP1B1 cDNA positions that are different between chimpanzee and human, and the corresponding alleles of the human, chimpanzee and gorilla reference genomes, and of the PanMap chimpanzees. (XLSX)

S1 Text Supporting Information. (DOCX)
