# Supplementary material for: Fire Usage and Ancient Hominin Detoxification Genes: Protective Ancestral Variants Dominate While Additional Derived Risk Variants Appear in Modern Humans
Source: PLoS One. 2016 Sep 21;11(9):e0161102. doi: 10.1371/journal.pone.0161102 (PMC5031311; doi:10.1371/journal.pone.0161102)
Supplement: S1 Table — (DOCX) [file pone.0161102.s001.docx]

*Genes and polymorphisms relevant for defence against toxic smoke components and food heating products analysed in this study.*

| **Gene Symbol** | **Gene Name** | **Polymorphism^1)^** | **Reported association of**  **reproduction-related**  **adverse effects**  **(literature references)** | | |
| --- | --- | --- | --- | --- | --- |
|  |  |  | **Female** | **Male** | **Biochemical level only** |
| AHR | Aromatic hydrocarbon receptor | p.Arg554Lys |  | [[1](#_ENREF_1)] |  |
|  |  | c.66-3946A>G |  | [[1](#_ENREF_1)] |  |
|  |  | p.Val381Ala^2)^ | [[2](#_ENREF_2)] |  | [[3](#_ENREF_3)] |
| AHRR | Ah receptor repressor | p.Pro189Ala |  | [[4](#_ENREF_4)] | [[5](#_ENREF_5)] |
| CAT | Catalase | c.-330C>T |  |  | [[6](#_ENREF_6)] |
| CYP1A1 | Cytochrome P450 1A1 | c.*1189T>C | [[7](#_ENREF_7)] | [[8](#_ENREF_8)] |  |
|  |  | c.-26-728C>T | [[9](#_ENREF_9)] |  |  |
|  |  | p.Ile462Val | [[10](#_ENREF_10)] |  |  |
| CYP1B1 | Cytochrome P450 1B1 | p.Leu432Val | [[11](#_ENREF_11)] |  | [[12](#_ENREF_12)] |
| EPHX1 | Epoxide hydrolase 1 | p.Tyr113His | [[9](#_ENREF_9)] |  |  |
|  |  | p.His139Arg | [[9](#_ENREF_9)] |  |  |
| EPHX2 | Epoxide hydrolase 2 | c.*93T>C |  | [[13](#_ENREF_13)] |  |
| ERCC1 | Excision Repair Cross-Complementing Rodent Repair Deficiency, Complementation Group 1 | c.*197G>T |  | [[14](#_ENREF_14)] |  |
| GSTA4 | Glutathione-S-transferase alpha 4 | c.415-48C>G | [[9](#_ENREF_9)] |  |  |
|  |  | c.139+176T>C | [[9](#_ENREF_9)] |  |  |
| GSTM1 | Glutathione-S-transferase mu 1 | Null | [[15](#_ENREF_15)] | [[16](#_ENREF_16)] |  |
| GSTP1 | Glutathione-S-transferase pi 1 | p.Ile105Val | [[9](#_ENREF_9)] |  |  |
|  |  | p.Ala114Val | [[9](#_ENREF_9)] |  |  |
|  |  | c.232+13C>A | [[9](#_ENREF_9)] |  |  |
| GSTT1 | Glutathione-S-transferase theta 1 | Null | [[9](#_ENREF_9), [15](#_ENREF_15)] |  |  |
| HIF1A | Hypoxia-inducible factor 1, alpha Subunit | c.1609-675C>A | [[9](#_ENREF_9)] |  |  |
| NAT1 | N-acetyl transferase 1 | c.*215A>T | [[17](#_ENREF_17)] |  |  |
|  |  | c.*222A>C | [[17](#_ENREF_17)] |  |  |
|  |  | p.Arg187Gln | [[18](#_ENREF_18)] |  | [[19](#_ENREF_19)] |
|  |  | p.Arg187Ter | [[18](#_ENREF_18)] |  | [[20](#_ENREF_20)] |
|  |  | p.Arg64Trp | [[18](#_ENREF_18)] |  | [[21](#_ENREF_21)] |
|  |  | p.Arg33Ter | [[18](#_ENREF_18)] |  | [[21](#_ENREF_21)] |
|  |  | p.Asp251Val | [[18](#_ENREF_18)] |  | [[21](#_ENREF_21)] |
| NAT2 | N-acetyl transferase 2 | p.Ile114Thr | [[22](#_ENREF_22), [23](#_ENREF_23)] |  | [[24](#_ENREF_24)] |
|  |  | p.Arg197Gln | [[9](#_ENREF_9), [22](#_ENREF_22), [23](#_ENREF_23)] |  |  |
| SOD2 | Superoxide dismutase 2 | p.Ala16Val | [[25](#_ENREF_25)] |  |  |
|  |  | c.*441G>A | [[25](#_ENREF_25)] |  |  |
| SULT1A1 | Sulfotransferase 1A1 | p.Arg213His | [[26](#_ENREF_26)] |  |  |
| UGT1A7 | UDP glucuronosyltransferase 1, polypeptide A7 | p.Asn129Lys / p.Arg131= / p.Arg131Gln | [[9](#_ENREF_9)] |  |  |
|  |  | p.Trp208Arg | [[9](#_ENREF_9)] |  |  |
| XPA1 | Xeroderma pigmentosum, complementation group A | c.-4A>G |  | [[14](#_ENREF_14)] |  |
|  |  | p.Arg156= |  | [[8](#_ENREF_8)] |  |

1. Nomenclature as recommended by the Human Genome Variation Society (<http://www.hgvs.org/mutnomen>, accessed February 1, 2016) [[27](#_ENREF_27)].
2. Not known in modern humans; no reference SNP code available in dbSNP.

**References**

1. Safarinejad MR, Shafiei N, Safarinejad S. Polymorphisms in Aryl Hydrocarbon Receptor Gene Are Associated With Idiopathic Male Factor Infertility. Reprod Sci. 2013;20(12):1423-32.

2. Pawlak AL, Strauss E, Florek E. Low activity of the ahr gene in the ahr dd C57BL congenic mice does not prevent the depression of birth rates observed as result of cigarette smoke exposure. Folia Histochem Cytobiol. 2001;39 Suppl 2:79-80.

3. Moriguchi T, Motohashi H, Hosoya T, Nakajima O, Takahashi S, Ohsako S, et al. Distinct response to dioxin in an arylhydrocarbon receptor (AHR)-humanized mouse. Proc Natl Acad Sci U S A. 2003;100(10):5652-7.

4. Merisalu A, Punab M, Altmäe S, Haller K, Tiido T, Peters M, et al. The contribution of genetic variations of aryl hydrocarbon receptor pathway genes to male factor infertility. Fertil Steril. 2007;88(4):854-9.

5. Hung W-T, Lambert GH, Huang P-W, Patterson Jr DG, Guo YL. Genetic susceptibility to dioxin-like chemicals’ induction of cytochrome P4501A2 in the human adult linked to specific AhRR polymorphism. Chemosphere. 2013;90(9):2358-64.

6. Schults MA, Chiu RK, Nagle PW, Wilms LC, Kleinjans JC, van Schooten FJ, et al. Genetic polymorphisms in catalase and CYP1B1 determine DNA adduct formation by benzo(a)pyrene ex vivo. Mutagenesis. 2013;28(2):181-5.

7. Suryanarayana V, Deenadayal M, Singh L. Association of CYP1A1 gene polymorphism with recurrent pregnancy loss in the South Indian population. Hum Reprod. 2004;19(11):2648-52.

8. Rubes J, Rybar R, Prinosilova P, Veznik Z, Chvatalova I, Solansky I, et al. Genetic polymorphisms influence the susceptibility of men to sperm DNA damage associated with exposure to air pollution. Mutat Res-Fundam Mol Mech Mutag. 2010;683(1):9-15.

9. Shi M, Christensen K, Weinberg CR, Romitti P, Bathum L, Lozada A, et al. Orofacial Cleft Risk Is Increased with Maternal Smoking and Specific Detoxification-Gene Variants. Am J Hum Genet. 2007;80(1):76-90.

10. Sram RJ, Binkova B, Dejmek J, Chvatalova I, Solansky I, Topinka J. Association of DNA adducts and genotypes with birth weight. Mutat Res. 2006;608(2):121-8.

11. Vidal JD, VandeVoort CA, Marcus CB, Lazarewicz NR, Conley AJ. In vitro exposure to environmental tobacco smoke induces CYP1B1 expression in human luteinized granulosa cells. Reprod Toxicol. 2006;22(4):731-7.

12. Helmig S, Wenzel S, Maxeiner H, Schneider J. CYP1B1 mRNA inducibility due to benzo(a)pyrene is modified by the CYP1B1 L432V gene polymorphism. Mutagenesis. 2014;29(4):237-40.

13. Qin Y, Han X, Peng Y, Shen R, Guo X, Cao L, et al. Genetic variants in epoxide hydrolases modify the risk of oligozoospermia and asthenospermia in Han-Chinese population. Gene. 2012;510(2):171-4.

14. Gu A, Ji G, Zhu P, Zhou Y, Fu G, Xia Y, et al. Nucleotide excision repair polymorphisms, polycyclic aromatic hydrocarbon exposure, and their effects on sperm deoxyribonucleic acid damage and male factor infertility. Fertil Steril. 2010;94(7):2620-5.e5.

15. Lammer EJ, Shaw GM, Iovannisci DM, Finnell RH. Maternal smoking, genetic variation of glutathione s-transferases, and risk for orofacial clefts. Epidemiology (Cambridge, Mass ). 2005;16(5):698-701.

16. Rubes J, Selevan SG, Sram RJ, Evenson DP, Perreault SD. GSTM1 genotype influences the susceptibility of men to sperm DNA damage associated with exposure to air pollution. Mutat Res-Fundam Mol Mech Mutag. 2007;625(1–2):20-8.

17. Lammer EJ, Shaw GM, Iovannisci DM, Van Waes J, Finnell RH. Maternal Smoking and the Risk of Orofacial Clefts: Susceptibility With NAT1 and NAT2 Polymorphisms. Epidemiology. 2004;15(2):150-6.

18. Carmichael SL, Shaw GM, Yang W, Iovannisci DM, Lammer E. Risk of limb deficiency defects associated with NAT1, NAT2, GSTT1, GSTM1, and NOS3 genetic variants, maternal smoking, and vitamin supplement intake. Am J Med Genet A. 2006;140A(18):1915-22.

19. Payton MA, Sim E. Genotyping human arylamine N-acetyltransferase type 1 (NAT1): the identification of two novel allelic variants. Biochem Pharmacol. 1998;55(3):361-6.

20. Hughes NC, Janezic SA, McQueen KL, Jewett MA, Castranio T, Bell DA, et al. Identification and characterization of variant alleles of human acetyltransferase NAT1 with defective function using p-aminosalicylate as an in-vivo and in-vitro probe. Pharmacogenetics. 1998;8(1):55-66.

21. Zhu Y, Hein DW. Functional effects of single nucleotide polymorphisms in the coding region of human N-acetyltransferase 1. Pharmacogenom J. 2008;8(5):339-48.

22. Hecht JT, Ester A, Scott A, Wise CA, Iovannisci DM, Lammer EJ, et al. NAT2 variation and idiopathic talipes equinovarus (clubfoot). Am J Med Genet A. 2007;143a(19):2285-91.

23. Sommer A, Blanton SH, Weymouth K, Alvarez C, Richards BS, Barnes D, et al. Smoking, the Xenobiotic Pathway, and Clubfoot. Birth Defects Res Part A: Clin Mol Teratol. 2011;91(1):20-8.

24. Fretland AJ, Leff MA, Doll MA, Hein DW. Functional characterization of human N-acetyltransferase 2 (NAT2) single nucleotide polymorphisms. Pharmacogenetics. 2001;11(3):207-15.

25. Giusti B, Vestrini A, Poggi C, Magi A, Pasquini E, Abbate R, et al. Genetic polymorphisms of antioxidant enzymes as risk factors for oxidative stress-associated complications in preterm infants. Free Radic Res. 2012;46(9):1130-9.

26. Wang L, Jin L, Liu J, Zhang Y, Yuan Y, Yi D, et al. Maternal genetic polymorphisms of phase II metabolic enzymes and the risk of fetal neural tube defects. Birth Defects Res Part A: Clin Mol Teratol. 2014;100(1):13-21.

27. den Dunnen JT, Antonarakis SE. Mutation nomenclature extensions and suggestions to describe complex mutations: a discussion. Hum Mutat. 2000;15(1):7-12.
