# Supplementary material for: Fire Usage and Ancient Hominin Detoxification Genes: Protective Ancestral Variants Dominate While Additional Derived Risk Variants Appear in Modern Humans
Source: PLoS One. 2016 Sep 21;11(9):e0161102. doi: 10.1371/journal.pone.0161102 (PMC5031311; doi:10.1371/journal.pone.0161102)
Supplement: S1 Text — (DOCX) [file pone.0161102.s005.docx]

**Supporting Information**

1. **Selection of relevant genes and polymorphisms**

Genes and polymorphisms of interest for the purpose of this study were selected from the epidemiological and biochemical-toxicological literature by applying the following three requirements:

1. A gene is considered relevant when encoding a protein with a well-established effect on the genotoxicity of smoke constituents, such as polycyclic aromatic hydrocarbons, polyhalogenated dibenzo-*p*-dioxins and dibenzofurans, and other aryl hydrocarbon receptor (AHR) agonists [[1-3](#_ENREF_1)], or food-heating products, such as PhIP and other heterocyclic amines (HCAs) [[4](#_ENREF_4)]. This effect is demonstrated by biochemical and/or toxicological mechanistic evidence, and could include, for example, influencing the detoxification or the bioactivation of these compounds.
2. In addition, polymorphisms for the selected gene have been reported in the epidemiological or biochemical literature that alter the protective efficiency of the encoded gene product against genotoxicity, and preferably also affect a toxic endpoint pertaining to reproductive success. If a study only reports the effect of a certain polymorphism on a relevant defence capability on the biochemical level, but does not explicitly include an analysis of the expected effects on human reproduction, results of that study are only included if it has been established that the particular polymorphism has an impact on another genotoxic endpoint, for example the risk of developing a certain form of cancer.
3. Exposure to tobacco smoke and/or food heating products was reported to modulate the risk of genotoxic effects, in particular adverse reproduction effects, associated with each genetic polymorphism meeting requirement 2.

We also searched the Gene Ontology (AmiGO 2) database [[5](#_ENREF_5)] for genes and gene products associated with smoke exposure, using the search terms “polycyclic hydrocarbon”, “dioxin”, “benzo[a]pyrene”, “smoke”, filtered for “Homo sapiens”. This approach did not generate relevant genes other than the ones identified by literature search according to the above-mentioned criteria.

S1 Table of the online Supporting Information (SI) lists the selected genes and polymorphisms, their reported association with the risk of adverse reproduction and genotoxic effects upon exposure to toxic smoke constituents and food heating products, and the corresponding literature references. Online S2 Table provides detailed genetic variant information for these polymorphisms as retrieved for the investigated Neanderthal, Denisovan, chimpanzee and gorilla genomes.

1. **Statistical aspects**

For the 35 detoxification gene loci studied, we observed that in 29 cases one or both ancient hominin species, Neanderthal and/or Denisovan, carried the low-risk allele. The six low-coverage Neanderthal genomes in all but one case displayed the same allele as was observed in the high-coverage Altai Neanderthal genome (S2 Table). Therefore the Altai Neanderthal may be considered representative of the group of seven Neanderthal genomes studied. The 52-fold coverage of the sequence data obtained from the Altai Neanderthal genome [[6](#_ENREF_6)], and the 30-fold coverage of the Denisovan genome [[7](#_ENREF_7)] allowed us to conclude that both genomes displayed only a single allele at 33 of these 35 loci, implying that they were homozygous for the observed allele. Only for the GSTM1 and GSTT1 wild-type/null polymorphism could the zygosity of the observed wild-type low-risk allele not be established.

To find out how biased towards predominance of the low-risk alleles the ancient hominins are, we determined the distribution of the number of low-/high-risk alleles within the present-day global population as represented by 2504 human genomes that have been analysed by the 1000 Genome Project Consortium [[8](#_ENREF_8)], and the position of the Altai Neanderthal and Denisovan within this distribution (Fig A). This frequency distribution shows that the probabilities of finding a contemporary human with an equally or more prominent predominance of the low-risk alleles as was observed in the Altai Neanderthal and Denisovan are 23.8% and 3.35%, respectively. Consequently, the probability of observing such a pair of individuals (with an equal or greater excess of low-risk alleles as the Altai Neanderthal and Denisovan, respectively) in the present-day population, is 0.238 × 0.0335 × 2 (because sampling order within this pair does not matter) × 100% = 1.59%.

In the oldest anatomically modern human genome available, that of an Upper Palaeolithic hunter-gatherer found at Ust’-Ishim in Siberia, we found a clear shift towards a higher number of loci carrying 1 or 2 high-risk alleles as compared to both the Altai Neanderthal and the Denisovan hominin genomes (see Fig 1 of the main article text). Fig A (below) illustrates, however, that at the allele level the difference is less distinct, because the Ust’-Ishim individual carries its 7 additional high-risk alleles at heterozygous loci (where both Altai Neanderthal and Denisovan are homozygous low-risk), whereas the high-risk alleles in the Altai Neanderthal and Denisovan are always in the homozygous state (S2 Table). As a result the Ust’-Ishim genome, with 18 high-risk alleles, again shows a marked increase as compared to the Denisovan, also with regard to the number of high-risk alleles, and is still substantially below the median of 21 high-risk alleles in the present-day population, but it is comparable to the Altai Neanderthal (Note that this is due to the fact that 3 polymorphisms of the 35 studied here were not covered by the 1000 Genomes variant data set; over the entire group of 35 loci the proportion of high-/low-risk alleles is 21/49 for Ust’-Ishim, 20/50 for the Altai Neanderthal, and 16/54 for the Denisovan).

Fig A. Distribution of the number of low-/high-risk alleles within the global population of the 1000 Genome Project and the relative position of the Altai Neanderthal and the Denisovan high-coverage genomes. The 1000 Genome Project data [[8](#_ENREF_8)] were retrieved from [[9](#_ENREF_9)]. Genotype data for the SNPs and the GSTM1 (esv3587154) and GSTT1 (esv3647425) deletion variants of S2 Table (SI) were categorised according to the number of low-/high-risk variants observed per individual using R [[10](#_ENREF_10)]. Three SNPs (rs2292596, rs56318881, and rs9282861) analysed in this study were not covered by the 1000 Genome Project variant data and were therefore not included in this frequency distribution. The average number of low-/high-risk alleles was 43/21 and overlapped with the median of this distribution. For each of the GSTM1 and GSTT1 loci in the ancient hominin genomes, being possibly homozygous or heterozygous low-risk (S2 Table), a contribution of 1 high-risk allele was conservatively counted in.


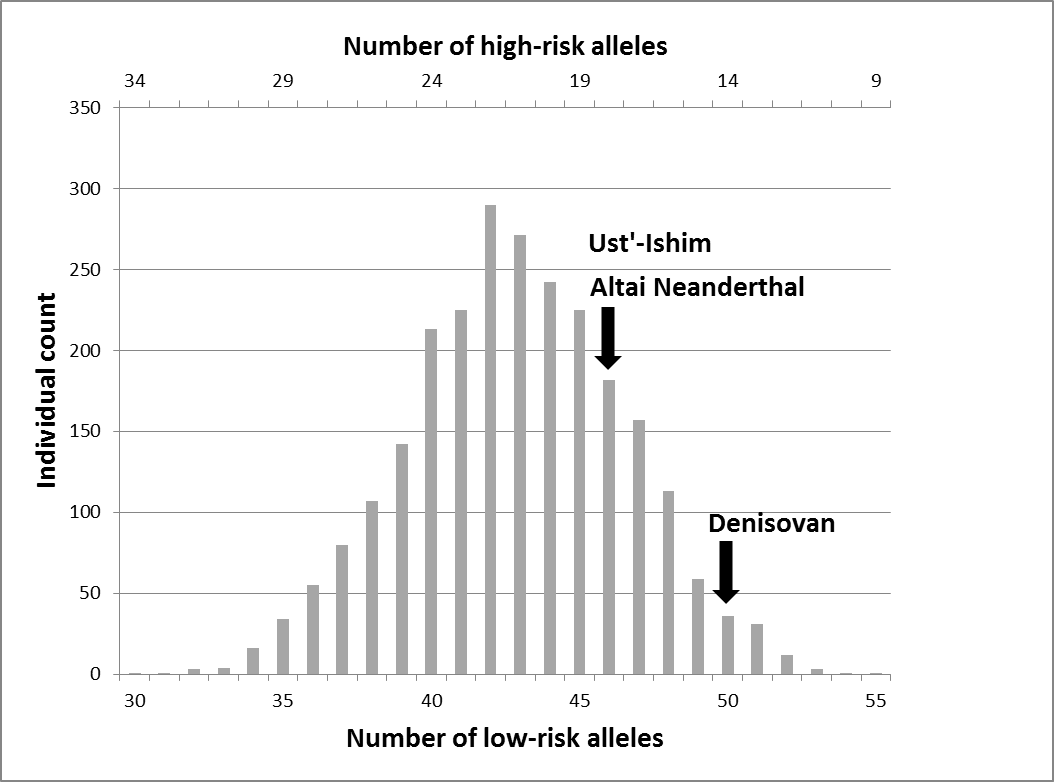


1. **Determination of genetic variants**

The genetic variants occurring in Neanderthal and Denisovan genomes were derived from next generation sequencing data in binary sequence alignment/map (BAM) format reported in the literature. These analyses included two high-coverage genomes, one from a Denisovan [[7](#_ENREF_7), [11](#_ENREF_11)] and one from a Neanderthal individual [[6](#_ENREF_6)], both found at Denisova cave in Siberia. When covered by these data sets, variant information was also retrieved from six low-coverage Neanderthal genomes derived from fossil bones found at the Feldhofer (prototype Neanderthal), Mezmaiskaya (Neanderthal infant), El Sidron (Sid1253), and Vindija archaeological sites (individuals Vi33.16, Vi33.25 and Vi33.26) [[12](#_ENREF_12)]. To visualize the Altai Neanderthal genomic sequences the BAM files of each chromosome [[13](#_ENREF_13)] were loaded into a custom track of the University of California, Santa Cruz (UCSC) Genome Browser using reference genome assembly GRCh37/hg19. The Denisovan genome sequences [[14](#_ENREF_14)] were determined using the “Denisova High-Coverage Sequence Reads” track integrated in the UCSC browser. Genetic variants of the low-coverage Neanderthal genomes were determined using the standard “Neandertal Sequence Reads” tracks of the NCBI36/hg18 version of the UCSC Genome Browser, which is based on published low-coverage sequence data [[12](#_ENREF_12)]. Variant calling was based on the predominant base observed over all sequence reads covering a particular position, applying at least 80% consensus as the lower confidence limit as an extra quality criterion for the high-coverage genomes only (Altai Neanderthal, Denisovan, and PanMap chimpanzees), and as the lower limit for homozygosity. Since the C to T and G to A transitions resulting from aging of ancient DNA occur relatively rarely and dispersed [[15](#_ENREF_15)], this approach also rules out variant calling mistakes due to this potential error source. For the low-coverage genomes S2 Table (online Supporting Information) lists the predominant base without applying quality requirements. The genetic variants occurring in pre-Holocene hunter-gatherers were derived from published genomes of two male individuals, dated to 45 and 24 thousand years ago (kya), respectively from Ust’-Ishim [[16](#_ENREF_16)] and Mal’ta [[17](#_ENREF_17)] in Siberia, and of Anzick-1, a male infant from North America dating to around 12.6 kya [[18](#_ENREF_18)]. The Holocene *Homo sapiens* genomes analysed include a Neolithic (NE1; 7.2 kya) and a Bronze Age (BR2; 3.2 kya) individual from Hungary [[19](#_ENREF_19)], an approximately 4,000 year old Palaeo-eskimo belonging to the Saqqaq Culture [[20](#_ENREF_20)], and a 100-year old Aboriginal Australian [[21](#_ENREF_21)]. These genomes were accessed by loading the corresponding BAM data files into a custom track of the UCSC Genome Browser (version GRCh37/hg19), except for the Hungarian genomes (NE1 and BR2), for which the genetic variants were retrieved from the NCBI Sequence Read Archive (Study Accession SRP039766) and analysed using their online alignment and sequence viewer tool [[22](#_ENREF_22)].

1. **Comparison with modern human and extant primate genetic variants**

All variant comparisons were performed using the UCSC Genome Browser. The chimpanzee variants were taken from the chimpanzee reference genome [[23](#_ENREF_23)] published by the Chimpanzee Sequencing and Analysis Consortium, and from the PanMap project reporting the genomic sequences of ten chimpanzee individuals [[24](#_ENREF_24)] of which the corresponding BAM files [[25](#_ENREF_25)] were read into a custom track of the UCSC browser. Gorilla variants were derived from the gorilla reference genome [[26](#_ENREF_26)]. Chimpanzee and gorilla reference genome variants were viewed using the “Multiz Alignment of 100 Vertebrates” track available in the UCSC browser. Allele frequencies of polymorphic sites among various modern human ethnic groups were retrieved from the 1000 Genomes Project data [[8](#_ENREF_8)] and complemented with data from the HapMap Project [[27](#_ENREF_27)] using the NCBI 1000 Genome Browser [[28](#_ENREF_28)], and the HapMap SNPs (release 27, merged Phase II+III genotypes) track of the UCSC Genome Browser, respectively. The variant data of the ethnic individuals were derived from Meyer *et al*. (2012) [[7](#_ENREF_7)], except for the Yoruba trio, which were retrieved from the March 2010 release of the 1000 Genomes Project [[29](#_ENREF_29)]. These data were accessed through the “Modern Humans Variant” and “Personal Variants Genome” tracks of the UCSC Genome Browser, respectively.

1. ***Genetic context of the gene polymorphisms of interest.***

Extrapolation of epidemiological data from modern humans to ancient hominins and chimpanzees would be compromised if, in those other species, the polymorphisms of interest should be embedded in a substantially different genetic context involving ancient haplotypes. However, with respect to ancient hominins it is known that the majority of sequence deviation from the modern human reference genome falls inside the variation between modern humans [[7](#_ENREF_7), [12](#_ENREF_12)]. This is reflected by the fact that of the known polymorphisms within the genes of interest, only one polymorphism (rs4987076 in *NAT1*) occurs in a catalogue of genetic variations that were derived ancient hominin-specific or modern human-specific [[6](#_ENREF_6)], which was however confined to differences for which the frequency of the allele in modern humans is higher than 90%. Therefore we carried out a comprehensive investigation of all coding region differences between modern humans and ancient hominins as well as chimpanzees for two model genes of key importance in the detoxification of PAHs, *CYP1A1* and *CYP1B1* (located on chromosome 15 and 2, respectively).

The complete protein-coding genomic sequence of the *CYP1A1* and *CYP1B1* genes of ancient hominins was extracted from the corresponding Variant Call Format (VCF) file [[30](#_ENREF_30), [31](#_ENREF_31)] using the coordinates of the RefSeq mRNA sequence (*CYP1A1*=NM_000499.3; *CYP1B1*=NM_000104.3) within the human reference genome (Version GRCh37/h19) as input for the 1000 Genomes Data Slicer web tool [[32](#_ENREF_32)]. Subsequently the data were imported into Excel (version 2010) and all positions that were different from the reference genome were filtered out to enable comprehensive determination of all differences occurring in translated sequences. Coding region boundaries were retrieved using the exon display utility within the Ensembl Genome Browser. The differences between the human (ENSG00000140465) and chimpanzee (ENSPTRG00000007280) *CYP1A1* cDNA sequences and the human (ENSG00000138061) and chimpanzee (ENSPTRG00000011843) *CYP1B1* cDNA sequences were determined using the orthologue alignment utility of the Ensembl Genome Browser [[33](#_ENREF_33), [34](#_ENREF_34)]. Subsequently, the UCSC Genome Browser was used to retrieve the corresponding variants of the CSAC 2.1.4/panTro4 chimpanzee reference genome [[23](#_ENREF_23)], the PanMap chimpanzees [[24](#_ENREF_24)] as well as the gorGor3.1/gorGor3 gorilla [[26](#_ENREF_26)] reference genome (S3 Table and S4 Table of the Supporting Information for *CYP1A1* and *CYP1B1*, respectively).

For *CYP1A1* we found a valine to methionine change at codon 482 (p.Val482Met) that was specific for the Denisovan and possibly Neanderthal individual Vi33.26 (based on a single read), and this derived Denisovan allele was observed in modern humans only in populations of European descent and at a very low frequency (below 1%, and therefore not mentioned by Prüfer et al [[6](#_ENREF_6)]). For *CYP1B1* we found a synonymous GTG>GTC mutation at Val243 which is a derived allele (chimpanzee has GTT and gorilla and various older primates have GTG) specifically occurring in all Neanderthal individuals (Denisovan has GTG). The Neanderthal GTC allele is extremely rare in all non-African modern humans (~0.1%), whereas, surprisingly, the Neanderthal allele occurs at much higher frequencies, up to 33%, in African Sub-Saharan populations. These observations confirm that differences in protein-coding sequences between ancient and modern human populations are also rare in these biotransformation enzyme genes, and, moreover, the existing differences lie within the variation spectrum observed in modern humans. These two cases corroborate our argument that, in the light of the established high overall similarity between ancient hominin and modern human genomes, extrapolation of epidemiological data obtained in modern human populations to ancient hominin genetic variants is justified. In the chimpanzee *CYP1A1* gene we found five sites with alleles that have not been reported in modern humans (S3 Table), and only one of these alleles leads to an amino acid substitution. For the *CYP1B1* gene we found seven such sites (S4 Table), six of which result in an amino acid change. Since these non-human alleles have not been tested in epidemiological studies, the effects of these variants remain to be determined.

Some of the observed low-risk variants might be inherited dependently as part of the same haplotype block, implying that a recombinatory event of these low-risk alleles with other variants within that block, if present in the population, would be relatively rare. However, for each of these SNPs in high linkage disequilibrium, a corresponding high-risk variant is found in present-day humans, and within each of these variant pairs the derived variant originated independently, which is the low-risk variant in an excessive majority of 26 cases. Therefore each of these 26 loci represents an independent case of the same characteristic pattern: a protective ancestral allele with an epidemiologically distinctive effect as compared to any other relevant SNPs, even when in strong linkage disequilibrium, is observed exclusively in ancient hominins, and mutated independently into a high-risk allele somewhere along the human evolutionary lineage, most likely after the split between the modern humans and ancient hominins. Therefore, our conclusion is independent of the haplotype structure of the loci investigated here.

1. **Determination of ancestral alleles**

To establish which is the ancestral allele we examined ten unrelated chimpanzee individuals from the PanMap Project [[24](#_ENREF_24)] in addition to the chimpanzee reference genome [[23](#_ENREF_23)], and to further exclude the possibility of erroneously assigning a chimpanzee-specific derived allele, we also compared to the gorilla reference genome [[26](#_ENREF_26)]. Within the group of 36 loci studied here, we found a discrepancy between the chimpanzee and the gorilla in only two cases, in which the gorilla did not match the chimpanzee nor the present-day human and ancient hominin genomes, pointing to a derived allele specifically occurring in the gorilla lineage. In those two cases we chose the chimpanzee allele as the most probable ancestral allele. We also confirmed that our ancestral allele assignment was consistent with the ancestral allele identified by the Enredo-Pecan-Ortheus multiple homologous sequence alignment pipeline [[35](#_ENREF_35)] as available within the Comparative Genomics section of the Ensemble Genome Browser (version GRCh37/Ensemble release 75) [[33](#_ENREF_33)]. The EPO-based allele was identical in all but three cases (GSTM1 WT/null, rs1057126, and rs1800975, where the EPO pipeline produced erroneous or no alignment output.

1. **Data summary**

In Table A (Altai Neanderthal) and Table B (Denisovan) the number of alleles observed in various categories is listed when classifying according to the high- and low-risk characteristic in combination with the ancestral or derived state as compared to chimpanzee.

| Table A | Number of studied gene loci per category  observed in the Altai Neanderthal | | | | |
| --- | --- | --- | --- | --- | --- |
|  | Low-/Low-risk | Low-/?-risk | Low-/High-risk | High-/High-risk | *Row total* |
| Ancestral/Ancestral | 22 |  |  | 6^2)^ | 28 |
| Ancestral/? |  | 2^1)^ |  |  | 2 |
| Ancestral/Derived |  |  | 1 in Vi33.26^3)^ |  | - |
| Derived/Derived | 2 |  |  | 3 | 5 |
| *Column total* | 24 | 2 | - | 9 | *Grand total* 35 |

- = not observed.

Hatched = illogical combination.

1. For the GSTM1 and GSTT1 WT>Null polymorphisms it is not possible to unequivocally distinguish zygosity on the basis of the available next generation sequencing data only.
2. Exclusive of AHR1 p.Val381Ala, for which the associated risk has been inferred, but never confirmed in a human context.
3. In only one instance an allele was observed in the six available low-coverage Neanderthal genomes [[12](#_ENREF_12)] that was different form the Altai Neanderthal: Vi33.26 from Vindija Cave is heterozygote high-/low-risk for p.Ile105Val in GSTP1 where Altai Neanderthal was homozygous high-risk. This case is indicated in this table but not included in the total numbers calculated for Altai Neanderthal.

| Table B |  | Number of studied gene loci per category  observed in the Denisovan | | | |
| --- | --- | --- | --- | --- | --- |
|  | Low-/Low-risk | Low-/?-risk | Low-/high-risk | High-/High-risk | *Row total* |
| Ancestral/Ancestral | 23 |  |  | 5^2)^ | 28 |
| Ancestral/? |  | 2^1)^ |  |  | 2 |
| Ancestral/Derived |  |  | - |  | - |
| Derived/Derived | 3 |  |  | 2 | 5 |
| *Column total* | 26 | 2 | - | 7 | *Grand total* 35 |

- = not observed.

Hatched = illogical combination.

1. For the GSTM1 and GSTT1 WT>Null polymorphisms it is not possible to unequivocally distinguish zygosity on the basis of the available next generation sequencing data only.
2. Exclusive of AHR1 p.Val381Ala, for which the associated risk has been inferred, but never confirmed in a human context.
3. **Predominance of low-risk alleles**

We sampled only two high coverage ancient hominin genomes (from the Altai Neanderthal and Denisovan) and six additional low-coverage Neanderthal genomes that are only partly informative for the 36 loci investigated in this study. A clear predominance of the low-risk, more protective alleles in the sample of ancient hominin genomes was observed over 35 relevant loci (Tables A, B, and S2 Table ; the risk associated with polymorphism p.Val381Ala in *AHR1* was inferred from animal data and never confirmed in a human context and therefore excluded from this analysis). However, for each single low-risk allele, even if observed as the only allele, there is a theoretical possibility that also the high-risk, or still another allele was present in the ancient hominin population that was missed by chance. Nevertheless, certain population-genetic principles enable to still make inferences regarding the likelihood that the observed alleles are predictive for the predominant allele present in the ancient populations. We found 21 loci (Table C) that were homozygous for the low risk-allele in both Altai Neanderthal and Denisovan and at which the low-risk allele is ancestral (Group A; the *GSTM1* and *GSTT1* deletion variants had to be excluded from this group, because their zygosity could not be unambiguously established if the wild-type allele was found). In those 21 cases the chance that the high-risk, derived allele is also present in the population at a considerable frequency is a priori very low for various reasons:

1. If present at all, the high risk allele is likely to be present at a relatively low frequency, since derived alleles tend to occur at much lower frequency than ancestral alleles [[36](#_ENREF_36)], except in rare cases of random drift towards substantial frequencies [[37](#_ENREF_37)], or when they have been under positive selection for a considerable number of generations [[36](#_ENREF_36)].
2. Instead, if adverse under contemporary conditions, it is more likely for a high-risk allele to be removed by purifying selection.
3. Moreover, Neanderthals have probably experienced population bottlenecks reducing their genetic diversity [[6](#_ENREF_6), [38](#_ENREF_38), [39](#_ENREF_39)] and also Denisovans have an unusually low genetic diversity and are exceptionally homozygous [[40](#_ENREF_40)], which precludes coexistence of multiple alleles at substantial frequencies, and therefore makes it less likely that another allele than the one observed would be prevalent in the population.
4. If the locus was found homozygous ancestral in two individuals of two different populations, it is even less likely that the derived allele would have reached a substantial frequency without appearing in our sample.

At 5 out of 35 loci (Table D) the high-risk allele is observed to be ancestral and homozygous in the Altai Neanderthal and Denisovan individual studied (Group B). Here, vice versa, reasons 1, 3 and 4 explain why the low-risk allele is unlikely to be present at a substantial frequency. Also prolonged positive selection, implying increased frequency, is improbable since the low-risk alleles have not been observed at these loci.

At the remaining 9 loci (Group C; Table E) a derived allele was actually present in one or both of the high-coverage ancient hominin genomes, or this possibility could not be excluded unequivocally (*GSTM1* and *GSTT1*). Therefore, these cases possibly represent one of the exceptions mentioned under reason 1 above. Hence it is less certain to what extent the actual observation is predictive of the proportion between the low- and high-risk allele in the respective ancient populations.

Nevertheless, if we consider the extreme case, that at the Group B and C loci the high-risk alleles would be the predominant allele (which is likely for Group B, and possible for Group C), the odds are still 21 against 14 in favour of the loci carrying the low-risk allele, instead of the actual proportion of 24 against 11, and 26 against 9 observed in Altai Neanderthal (Table A) and Denisovan (Table B), respectively. Altogether we conclude that the presented genomic data strongly suggest that, within the group of 35 detoxification enzyme polymorphisms studied here, the low-risk, more efficient detoxification variants are predominant over the high-risk, less efficient variants in Neanderthals and Denisovans.

1. **Appearance of new derived high-risk alleles in Ust’-Ishim and contemporary humans**

When compared to the contemporary global human population studied by the 1000 Genomes Project Consortium [[8](#_ENREF_8)], the Ust’-Ishim individual with 15 loci carrying 1 or 2 high-risk alleles (over the available data) is still slightly below the average and the median of the present-day human population, whereas the Altai Neanderthal (10 such loci) and Denisovan (8 such loci) are at the far and extreme end of the distribution, respectively (see Fig 1 in the main article text). This demonstrates that modern humans have a significantly higher number of loci carrying a high-risk allele than Neanderthals and Denisovans (see also section B) and strongly suggests that this increase already started in early Upper Palaeolithic hunter-gatherers 45,000 year ago. On the level of the number of high-risk alleles, however, changes are less distinct.

Comparing the Ust’-Ishim genome to that of Neanderthal and Denisovan hominins displays an additional heterozygous high-risk allele for 8 out of the 21 Group A loci in the former. This implies that these additional high-risk alleles probably had reached a substantial frequency in the Ust’-Ishim population, otherwise the chances would be low to actually observe a heterozygote [[41](#_ENREF_41)]. Accordingly, this result is suggesting an increase in number and/or frequency of the high-risk alleles within the Group A loci [[41](#_ENREF_41)].

The opposite was also observed: At 5 loci (Group B) both Altai Neanderthal and Denisovan were homozygous for the high-risk allele being the ancestral allele (S2 Table). At 2 out of these 5 loci Ust’-Ishim was found homozygous for the low-risk allele, being a newly derived allele as compared to chimpanzee.

For the Group C loci (with a derived allele in one or both ancient genomes) the allele actually observed in the ancient hominin genomes is, as pointed out above, less predictive of the major allele in the population, and therefore it is more difficult to draw a definitive conclusion regarding a change in number or frequency in Ust’-Ishim. The data however suggest a change at 3 of those loci, where the high-risk allele is derived and appears in one of the ancient genomes, whereas it is not observed at all in Ust’-Ishim. This suggests that the derived allele only originated in the ancient hominin lineage, and Ust’-Ishim has only the ancestral, low-risk allele and could be missing the high-risk allele at these 3 loci. In addition, within Group C at 1 locus (p.Trp208Arg in *UGT1A7*; S2 Table) both Altai Neanderthal and Denisovan are homozygous for the derived, high-risk allele, whereas the low-risk variant, was also observed in Ust’-Ishim. This situation suggests that in the ancient hominin lineage, the derived, high-risk allele became the major allele or reached fixation, whereas the Ust’-Ishim individual retained also the ancestral, low-risk allele next to the high-risk allele.

In summary, as compared to the two ancient hominin individuals studied and in consecutive order as described above, we observe in the Ust’-Ishim individual a probable gain of 8 high-risk alleles at the Group A loci, a possible gain of 2 low-risk alleles at the Group B loci, and a possible gain of 1 other low-risk allele, and a possible loss of 3 high-risk alleles at the Group C loci, resulting in a high-/low-risk allele proportion over all 35 loci studied of 21/49 for Ust’-Ishim, an almost similar 20/50 proportion for the Altai Neanderthal, and a markedly lower 16/54 proportion in the Denisovan. Altogether, it appears difficult to weigh all the observed changes against each other. Therefore it is not possible to draw a definitive conclusion in terms of increase in numbers or frequencies of the high risk alleles over the entire set of 35 SNPs, although the results suggest a small increase. However, altogether, the data clearly point to an increase in the number of loci carrying 1 or 2 high-risk alleles due to new appearances and/or increase in frequency of the high-risk alleles over the 21 Group A loci. Increased frequency is supported by the fact that the high-risk variants often attain substantial allele frequencies in contemporary humans, and in 11 cases even became the major allele (Tables C-E; S2 Table), with frequencies up to the 80% range. Even though the gained high-risk allele is still found in the heterozygous state in Ust’-Ishim at all 8 particular loci in Group A, heterozygosity in general implies coexistence of both alleles at considerable frequencies; the more unbalanced the frequencies the lower the chance that a heterozygote will be generated [[41](#_ENREF_41)].

*Table C. Global allele frequencies in extant modern humans for the subgroup of loci (Group A, homozygous ancestral and low-risk in both Altai Neanderthal and Denisovan) at which new appearances and/or increase in frequency of the high-risk alleles along the human lineage are highly likely, in particular when becoming the major allele (grey highlighting).*

| **Gene Symbol** | **Gene Name** | **Polymorphism** | **dbSNP ID** | **Global allele frequency (dbSNP)** | |
| --- | --- | --- | --- | --- | --- |
|  |  |  |  | **Low-risk** | **High-risk** |
| AHR1 | Aromatic hydrocarbon receptor | p.Arg554Lys | rs2066853 | **A** | **G** |
|  |  |  |  | 0.2708 | 0.7292 |
|  |  | c.66-3946A>G | rs2282885^6)^ | **T** | **C** |
|  |  |  |  | 0.8061 | 0.1939 |
| AHRR1^13^ | Ah receptor repressor | p.Pro189Ala | rs2292596 | **G** | **C** |
|  |  |  |  | 0.35282^#)^ | 0.64718^#)^ |
| CYP1A1 | Cytochrome P450 1A1 | c.*1189T>C | rs4646903 | **T** | **C** |
|  |  |  |  | 0.7069 | 0.2931 |
|  |  | p.Ile462Val | rs1048943 | **A** | **G** |
|  |  |  |  | 0.8666 | 0.1334 |
| CYP1B1 | Cytochrome P450 1B1 | p.Leu432Val | rs1056836 | **G** | **C** |
|  |  |  |  | 0.3852 | 0.6148 |
| EPHX1 | Epoxide hydrolase 1 | p.Tyr113His | rs1051740 | **T** | **C** |
|  |  |  |  | 0.6867 | 0.3133 |
| EPHX2 | Epoxide hydrolase 2 | c.*93T>C | rs1042064 | **C** | **T** |
|  |  |  |  | 0.4187 | 0.5813 |
| GSTP1 | Glutathione-S-transferase pi 1 | p.Ala114Val | rs1138272 | **C** | **T** |
|  |  |  |  | 0.9665 | 0.0335 |
|  |  | c.232+13C>A | rs762803 | **C** | **A** |
|  |  |  |  | 0.6881 | 0.3119 |
| NAT1 | N-acetyl transferase 1 | p.Arg187Gln | rs4986782 | **G** | **A** |
|  |  |  |  | 0.9944 | 0.0056 |
|  |  | p.Arg187Ter | rs5030839 | **C** | **T** |
|  |  |  |  | 0.9972 | 0.0028 |
|  |  | p.Arg64Trp | rs56379106 | **C** | **T** |
|  |  |  |  | 0.9988 | 0.0012 |
|  |  | p.Arg33Ter | rs56318881 | **C** | **T** |
|  |  |  |  | 0.99995882 | 0.00004118*^)^ |
|  |  | p.Asp251Val | rs56172717 | **A** | **T** |
|  |  |  |  | 0.9988 | 0.0012 |
| NAT2 | N-acetyl transferase 2 | p.Ile114Thr | rs1801280 | **T** | **C** |
|  |  |  |  | 0.7073 | 0.2927 |
|  |  | p.Arg197Gln | rs1799930 | **G** | **A** |
|  |  |  |  | 0.735 | 0.265 |
| CAT1 | Catalase | c.-330C>T | rs1001179^6)^ | **G** | **A** |
|  |  |  |  | 0.8744 | 0.1256 |
| SOD2 | Superoxide dismutase 2 | p.Ala16Val | rs4880 | **C** | **T** |
|  |  |  |  | 0.4107 | 0.5893 |
|  |  | c.*441G>A | rs5746136 | **G** | **A** |
|  |  |  |  | 0.6943 | 0.3057 |
| XPA1 | Xeroderma pigmentosum, | c.-4A>G | rs1800975 | **G** | **A** |
|  | complementation group A |  |  | 0.6464 | 0.3536 |

#) Data from the UCSC Genome Browser.

*) Based on a single submitted SNP (ss1689107584).

*Table D. Global allele frequencies in extant modern humans for the Group B loci (homozygous ancestral and high-risk in both Altai Neanderthal and Denisovan; grey highlighting indicates when the high-risk variant is the major allele).*

| **Gene Symbol** | **Gene Name** | **Polymorphism** | **dbSNP ID** | **Global allele frequency (dbSNP)** | |
| --- | --- | --- | --- | --- | --- |
|  |  |  |  | **Low risk** | **High risk** |
| CYP1A1 | Cytochrome P450 1A1 | c.-26-728C>T | rs4646421 | T | C |
|  |  |  |  | 0.3241 | 0.6759 |
| NAT1 | N-acetyl transferase 1 | c.*222A>C | rs15561 | C | A |
|  |  |  |  | 0.5627 | 0.4373 |
| SULT1A1 | Sulfotransferase 1A1 | p.Arg213His | rs9282861 | A | G |
|  |  |  |  | 0.18668^#)^ | 0.81332^#)^ |
| UGT1A7 | UDP glucuronosyltransferase 1, polypeptide A7 | p.Asn129Lys/ p.Arg131=/ p.Arg131Gln | rs17868323/ rs17863778/ rs17868324 | T/C/G | G/A/A |
|  |  |  |  | 0.4237 | 0.5763 |
| HIF1A1 | Hypoxia-inducible factor 1, alpha Subunit | c.1609-675C>A | rs2301113 | A | C |
|  |  |  |  | 0.53 | 0.47 |

#) Data from the UCSC Genome Browser.

*Table E. Global allele frequencies in extant modern humans for the Group C loci (Altai Neanderthal and/or Denisovan carry a derived allele; grey highlighting indicates when the high-risk variant is the major allele).*

| **Gene Symbol** | **Gene Name** | **Polymorphism^1)^** | **dbSNP ID** | **Global allele frequency (dbSNP)** | |
| --- | --- | --- | --- | --- | --- |
|  |  |  |  | **Low-risk** | **High-risk** |
| EPHX1 | Epoxide hydrolase 1 | p.His139Arg | rs2234922 | **A** | **G** |
|  |  |  |  | 0.7845 | 0.2155 |
| GSTA4 | Glutathione-S-transferase alpha 4 | c.415-48C>G | rs316133 | **G** | **C** |
|  |  |  |  | 0.4249 | >0.4249^$)^ |
|  |  | c.139+176T>C | rs3756980 | **T** | **C** |
|  |  |  |  | 0.8548 | 0.1452 |
| GSTM1 | Glutathione-S-transferase mu 1 | WT > Null | - | **WT** | **Null** |
|  |  |  |  | 0.352^%)^ | 0.648^%)^ |
| GSTP1 | Glutathione-S-transferase pi 1 | p.Ile105Val | rs1695 | **A** | **G** |
|  |  |  |  | 0.6474 | 0.3526 |
| GSTT1 | Glutathione-S-transferase theta 1 | WT > Null | - | **WT** | **Null** |
|  |  |  |  | 0.497^%)^ | 0.503^%)^ |
| NAT1 | N-acetyl transferase 1 | c.*215A>T | rs1057126^10)^ | **T** | **A** |
|  |  |  |  | 0.5994 | 0.4006 |
| UGT1A7 | UDP glucuronosyltransferase 1, | p.Trp208Arg | rs11692021 | **T** | **C** |
|  | polypeptide A7 |  |  | 0.7023 | 0.2977 |
| ERCC1 | Excision Repair Cross-Complementing | c.*197G>T | rs3212986 | **G** | **T** |
|  | Rodent Repair Deficiency, Complementation Group 1 |  |  | 0.7049 | 0.2951 |

%) Phase 1 data of the 1,000 Genomes Project [[42](#_ENREF_42)].

$) There are three alleles known, so the minor allele frequency (MAF) reported in dbSNP is the allele with the second highest frequency, implying that the major allele must have a higher frequency with MAF as lower and (1-MAF) as upper boundary.

1. **References**

1. Freeman DJ, Cattell FCR. Woodburning as a source of atmospheric polycyclic aromatic hydrocarbons. Environ Sci Technol. 1990;24(10):1581-5.

2. Schatowitz B, Brandt G, Gafner F, Schlumpf E, Bühler R, Hasler P, et al. Dioxin emissions from wood combustion. Chemosphere. 1994;29(9–11):2005-13.

3. Zhong Y, Carmella SG, Upadhyaya P, Hochalter JB, Rauch D, Oliver A, et al. Immediate consequences of cigarette smoking: Rapid formation of polycyclic aromatic hydrocarbon diol epoxides. Chem Res Toxicol. 2011;24(2):246-52.

4. Skog KI, Johansson MAE, Jägerstad MI. Carcinogenic heterocyclic amines in model systems and cooked foods: A review on formation, occurrence and intake. Food Chem Toxicol. 1998;36(9–10):879-96.

5. The Gene Ontology Consortium. AmiGO 2, version: 2.3.2 (amigo2b) [Internet]. The Gene Ontology 2016 [updated 2016 May 23; cited 2016 June 1]. Available from: <http://amigo.geneontology.org/amigo/landing>.

6. Prüfer K, Racimo F, Patterson N, Jay F, Sankararaman S, Sawyer S, et al. The complete genome sequence of a Neanderthal from the Altai Mountains. Nature. 2014;505(7481):43-9.

7. Meyer M, Kircher M, Gansauge M-T, Li H, Racimo F, Mallick S, et al. A High-Coverage Genome Sequence from an Archaic Denisovan Individual. Science. 2012;338(6104):222-6.

8. The 1000 Genomes Project Consortium. A global reference for human genetic variation. Nature. 2015;526(7571):68-74.

9. The 1000 Genome Project Consortium. FTP directory /vol1/ftp/release/20130502/ at ftp.1000genomes.ebi.ac.uk [Internet]. The 1000 Genome Project Consortium; 2016 [updated 2015 May 27; cited 2016 June 1]. Latest Phase 3 release of the 1000 Genomes Project data. Available from: <ftp://ftp.1000genomes.ebi.ac.uk/vol1/ftp/release/20130502/>.

10. R Core Team. R: A Language and Environment for Statistical Computing Vienna, Austria: R Foundation for Statistical Computing; 2015. Available from: <https://www.R-project.org>.

11. Reich D, Green RE, Kircher M, Krause J, Patterson N, Durand EY, et al. Genetic history of an archaic hominin group from Denisova Cave in Siberia. Nature. 2010;468(7327):1053-60.

12. Green RE, Krause J, Briggs AW, Maricic T, Stenzel U, Kircher M, et al. A Draft Sequence of the Neandertal Genome. Science. 2010;328(5979):710-22.

13. Index of /neandertal/altai/AltaiNeandertal/bam [Internet]. Leipzig: Max Planck Institute for Evolutionary Anthropology; 2013 [updated 2013 April 12; cited 2016 Febr. 1]. BAM files of Altai Neanderthal genomic sequences mapped to human reference genome hg19. Available from: <http://cdna.eva.mpg.de/neandertal/altai/AltaiNeandertal/bam>.

14. Index of /denisova/alignments [Internet]. Leipzig: Max Planck Institute for Evolutionary Anthropology; 2012 [updated 2012 Febr. 6; cited 2016 Febr. 1]. BAM files of Denisovan genomic sequences mapped to human reference genome hg19. Available from: <http://cdna.eva.mpg.de/denisova/alignments>.

15. Briggs AW, Stenzel U, Johnson PLF, Green RE, Kelso J, Prüfer K, et al. Patterns of damage in genomic DNA sequences from a Neandertal. Proc Natl Acad Sci U S A. 2007;104(37):14616-21.

16. Fu Q, Li H, Moorjani P, Jay F, Slepchenko SM, Bondarev AA, et al. Genome sequence of a 45,000-year-old modern human from western Siberia. Nature. 2014;514(7523):445-+.

17. Raghavan M, Skoglund P, Graf KE, Metspalu M, Albrechtsen A, Moltke I, et al. Upper Palaeolithic Siberian genome reveals dual ancestry of Native Americans. Nature. 2014;505(7481):87-91.

18. Rasmussen M, Anzick SL, Waters MR, Skoglund P, DeGiorgio M, Stafford Jr TW, et al. The genome of a Late Pleistocene human from a Clovis burial site in western Montana. Nature. 2014;506(7487):225-9.

19. Gamba C, Jones ER, Teasdale MD, McLaughlin RL, Gonzalez-Fortes G, Mattiangeli V, et al. Genome flux and stasis in a five millennium transect of European prehistory. Nat Commun. 2014;5.

20. Rasmussen M, Li Y, Lindgreen S, Pedersen JS, Albrechtsen A, Moltke I, et al. Ancient human genome sequence of an extinct Palaeo-Eskimo. Nature. 2010;463(7282):757-62.

21. Rasmussen M, Guo X, Wang Y, Lohmueller KE, Rasmussen S, Albrechtsen A, et al. An Aboriginal Australian Genome Reveals Separate Human Dispersals into Asia. Science. 2011;334:94-8.

22. Sequence Read Archive, Study Accession SRP039766 [Internet]. National Center for Biotechnology Information; 2016 [updated 2016 Jan. 8; cited 2016 Febr. 1]. Ancient Hungarian Genomes. Available from: <http://trace.ncbi.nlm.nih.gov/Traces/sra/sra.cgi?study=SRP039766>.

23. The Chimpanzee Sequencing and Analysis Consortium. Initial sequence of the chimpanzee genome and comparison with the human genome. Nature. 2005;437(7055):69-87.

24. Auton A, Fledel-Alon A, Pfeifer S, Venn O, Ségurel L, Street T, et al. A Fine-Scale Chimpanzee Genetic Map from Population Sequencing. Science. 2012;336(6078):193-8.

25. Pfeifer S. FTP directory /panMap/BAMs/HG18 at birch.well.ox.ac.uk [Internet]. PanMap Project; 2012 [updated 25-9-2012; cited 2016 Febr. 1]. The BAM files for 10 chimpanzees with reads aligned to the human reference sequence hg18. Available from: <ftp://birch.well.ox.ac.uk/panMap/BAMs/HG18>.

26. Scally A, Dutheil JY, Hillier LW, Jordan GE, Goodhead I, Herrero J, et al. Insights into hominid evolution from the gorilla genome sequence. Nature. 2012;483(7388):169-75.

27. The International HapMap 3 Consortium. Integrating common and rare genetic variation in diverse human populations. Nature. 2010;467(7311):52-8.

28. 1000 Genomes Browser, Phase 3, version 3.5 [Internet]. Bethesda (MD): National Center for Biotechnology Information, U.S. National Library of Medicine 2015 [updated 2015 Sept. 29; cited 2016 Febr. 1]. Data of the 1000 Genomes Project, phase 3. Available from: <http://www.ncbi.nlm.nih.gov/variation/tools/1000genomes/>.

29. The 1000 Genomes Project Consortium. A map of human genome variation from population-scale sequencing. Nature. 2010;467(7319):1061-73.

30. Index of /denisova/VCF/hg19_1000g [Internet]. Leizig: Max Planck Institute for Evolutionary Anthropology; 2012 [updated 2012 Febr. 9; cited 2016 Febr. 1]. VCF files of Denisovan genomic sequences. Available from: <http://cdna.eva.mpg.de/denisova/VCF/hg19_1000g>.

31. Index of /neandertal/altai/AltaiNeandertal/VCF [Internet]. Leipzig: Max Planck Institute for Evolutionary Anthropology; 2013 [updated 2013 May 23; cited 2016 Febr. 1]. VCF files of Altai Neanderthal genomic sequences. Available from: <http://cdna.eva.mpg.de/neandertal/altai/AltaiNeandertal/VCF>.

32. Data Slicer [Internet]. 1000 Genomes; c2008-2012 [cited 2016 Febr. 1]. Interface to subsections of either vcf or bam files based on genomic coordinates. Available from: <http://www.1000genomes.org/data-slicer>.

33. Human Genome assembly GRCh37.p13, Ensemble release 83 [Internet]. Hinxton: WTSI / EMBL-EBI; 2015 [updated 2015 Dec. ; cited 2016 Febr. 1]. Gemome browser with orthologue alignment utility. Available from: <http://grch37.ensembl.org/Homo_sapiens/Info/Index>.

34. Cunningham F, Amode MR, Barrell D, Beal K, Billis K, Brent S, et al. Ensembl 2015. Nucleic Acids Res. 2015;43(D1):D662-D9.

35. Paten B, Herrero J, Fitzgerald S, Beal K, Flicek P, Holmes I, et al. Genome-wide nucleotide-level mammalian ancestor reconstruction. Genome Res. 2008;18(11):1829-43.

36. Sabeti PC, Schaffner SF, Fry B, Lohmueller J, Varilly P, Shamovsky O, et al. Positive Natural Selection in the Human Lineage. Science. 2006;312(5780):1614-20.

37. Hartl DL, Clark AG. Molecular Population Genetics. In: Principles of Population Genetics. Sunderland, MA, USA: Sinauer Associates Inc.; 1997. p. 315-95.

38. Sánchez-Quinto F, Lalueza-Fox C. Almost 20 years of Neanderthal palaeogenetics: adaptation, admixture, diversity, demography and extinction. Philos Trans R Soc Lond B Biol Sci. 2014;370(1660).

39. Bocquet-Appel J-P, Degioanni A. Neanderthal Demographic Estimates. Curr Anthrop. 2013;54(S8):S202-S13.

40. Meyer M, Kircher M, Gansauge M-T, Li H, Racimo F, Mallick S, et al. A High-Coverage Genome Sequence from an Archaic Denisovan Individual. Science. 2012.

41. Hartl DL, Clark AG. Organization of Genetic Variation. In: Principles of Population Genetics. Sunderland, MA, USA: Sinauer Associates Inc.; 1997. p. 71-109.

42. Karaca S, Karaca M, Cesuroglu T, Erge S, Polimanti R. GSTM1, GSTP1, and GSTT1 genetic variability in Turkish and worldwide populations. Am J Hum Biol. 2015;27(3):310-6.
